# Turning over on sticky balls: preparation and catalytic studies of surface-functionalized TiO_2_ nanoparticles†

**DOI:** 10.1039/d0ra09319j

**Published:** 2021-01-29

**Authors:** Sven A. Freimann, Alessandro Prescimone, Catherine E. Housecroft, Edwin C. Constable

**Affiliations:** Department of Chemistry, University of Basel BPR 1096, Mattenstrasse 24a CH-4058 Basel Switzerland edwin.constable@unibas.ch

## Abstract

We have investigated the reactivity of rhodium(iii) complex-functionalized TiO_2_ nanoparticles and demonstrate a proof-of-principle study of their catalytic activity in an alcohol oxidation carried out under aqueous conditions water in air. TiO_2_ nanoparticles (NPs) have been treated with (4-([2,2′:6′,2′′-terpyridin]-4′-yl)phenyl)phosphonic acid, 1, to give the functionalized NPs (1)@TiO_2_. Reaction between (1)@TiO_2_ NPs and either RhCl_3_·3H_2_O or [Rh_2_(μ-OAc)_4_(H_2_O)_2_] produced the rhodium(iii) complex-functionalized NPs Rh(1)_2_@TiO_2_. The functionalized NPs were characterized using thermogravimetric analysis (TGA), matrix-assisted laser desorption ionization (MALDI) mass spectrometry, ^1^H NMR and FT-IR spectroscopies; the single crystal structures of [Rh(1)_2_][NO_3_]_3_·1.25[H_3_O][NO_3_]·2.75H_2_O and of a phosphonate ester derivative were determined. ^1^H NMR spectroscopy was used to follow the reaction kinetics and to assess the recyclability of the NP-supported catalyst. The catalytic activity of the Rh(1)_2_@TiO_2_ NPs was compared to that of a homogeneous system containing [Rh(1)_2_]^3+^, confirming that no catalytic activity was lost upon surface-binding. Rh(1)_2_@TiO_2_ NPs were able to withstand reaction temperatures of up to 100 °C for 24 days without degradation.

## Abstract 2

A proof-of-principle investigation of the reactivity of functionalized NPs Rh(1)_2_@TiO_2_ (1 = (4-([2,2′:6′,2′′-terpyridin]-4′-yl)phenyl)phosphonic acid, 1) is reported, using their catalytic activity in an alcohol oxidation in aqueous conditions water.

## Graphical abstract

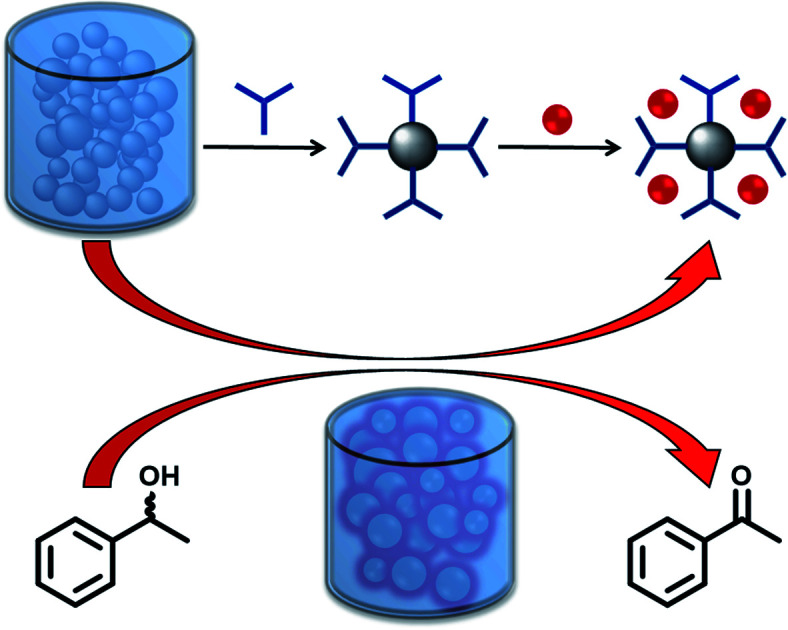

## Introduction

In general, catalytic processes are categorized as homogeneous or heterogeneous. Homogeneous catalysts have the advantage that all of the catalytic centres are potentially active, whereas in heterogeneous catalysts only the surface catalytic sites of solid phases are active as interior sites are inactive.^1^ With solid phase heterogeneous catalysts, reaction only takes place at the interface rather than in the bulk of the reactant volume. On the other hand, it is often easier to separate the products of heterogeneous catalysis from the spent catalyst than in the case of homogeneous catalysis.^2,3^ Heterogeneous catalysts also have significant benefits in terms of catalyst recovery, a feature gaining increasing environmental and economic importance.

Nanoparticle (NP) immobilized catalysts have the potential of bridging the gap between these two extreme types of catalysis. Firstly, NPs exhibit greater surface-to-volume ratios than bulk heterogeneous catalysts and the catalyst loading capacity, catalytic activity and turnover will be enhanced, in particular the catalytic site-to-volume ratio will be high.^4^ Secondly, the ability to disperse NPs in solution gives some of the benefits of homogeneous catalysts. NP-supported catalysts have attracted great interest and offer already outstanding diversity and range in the chemical and pharmaceutical field.^5–7^

The advantages of NP supported catalysts have been demonstrated for a number of key reactions. For the Suzuki–Miyaura coupling reaction, palladium-decorated benzene-1,2-diamine-functionalized Fe_3_O_4_/SiO_2_ magnetic NPs were utilized and showed excellent yields within short reaction times.^1^ Gold NP-supported ruthenium catalysts have been used for ring-opening metathesis polymerization of bicyclo[2.2.1]hept-2-ene (norbornene) and show a higher activity than the unsupported counterparts.^8^ Nickel NP-based catalysts were active in the steam–reformation reaction of methane showing excellent conversion, H_2_ selectivity and thermal stability.^9^ These reactions often make use of valuable and rare elements that cannot be efficiently recovered in the case of homogeneous catalysis. Recyclability of a catalyst is essential and conventional heterogeneous catalysts have the benefit that they can be easily recovered compared to homogeneous catalysts. Dispersed NPs are more challenging to separate than conventional heterogeneous catalysts but the recoverability is still greater than with most homogenous catalysts. Furthermore, NPs can be modified to boost recoverability by introducing additional characteristics such as magnetic properties.

The benefits of NPs are well-established in the oxidation of alcohols, a key step in the synthesis of many organic compounds.^7^ The transformation of alcohols to aldehydes, ketones or carboxylic acids generally needs stoichiometric quantities of hazardous, environmentally damaging and toxic oxidants such as chromium trioxide, dichromate, permanganate and chromic acid.^10^

**Scheme 1:**
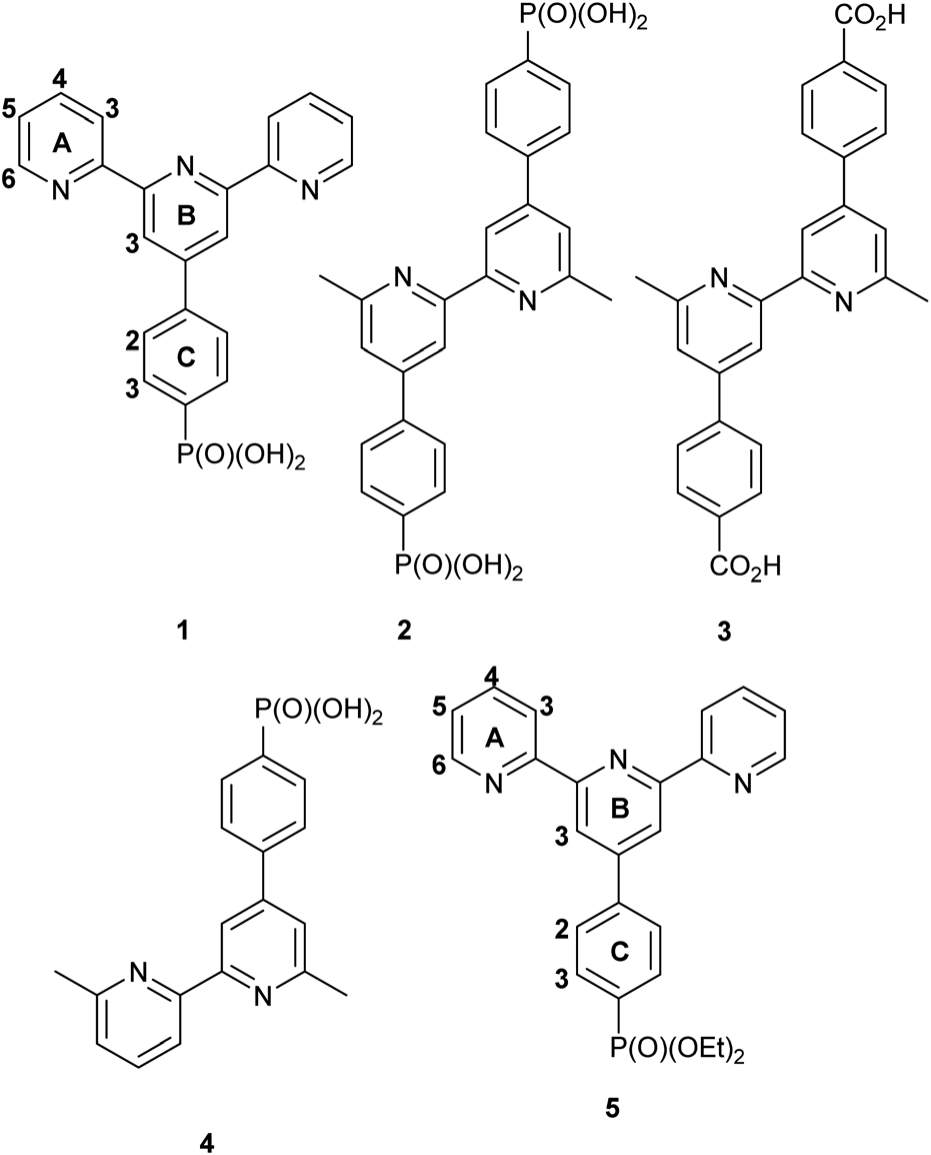
Structures of compounds 1–5.

We have recently demonstrated^11^ the functionalization of TiO_2_ NPs with ligands 1, 2, 3 and 4 (see Scheme 1) which bind to the metal oxide surface through their phosphonate or phosphonic acid groups and we were interested in investigating the activity of complexes incorporating these surface-bound ligands. We report here the assembly of surface-bound rhodium complexes of ligand 1 related to [Rh_2_Cl_2_(μ-OAc)(tpy)_2_]^+^ reported by Wang *et al.*^12^ We compare the catalytic behaviour of functionalized TiO_2_ NPs to the homogeneous catalytic performance of [Rh(1)_2_]Cl_3_ and [Rh(5)_2_][PF_6_]_3_. We have also investigated the kinetics of these reactions, the recyclability of the NP catalyst over 5 cycles, and the influence of the concentration of base on the catalytic activity of the functionalized nanoparticles.

## Experimental

### General

Instrumentation details are given in the ESI.†

Ligands 1 and 5 (Scheme 1),^13^ were prepared according to the literature and their spectroscopic data matched those previously reported. TiO_2_ NP activation and functionalization with ligand 1 were carried out according to our previously published procedure.^11^ MALDI mass spectra were recorded using α-cyano-4-hydroxycinnamic acid (CHCA) as the matrix.

[Rh_2_(μ-OAc)_4_(H_2_O)_2_] and *rac*-(1*R*)-1-phenylethanol were purchased from Acros Organics and Sigma Aldrich respectively. RhCl_3_·3H_2_O was purchased from Johnson Mathey, Materials Technology UK. Microwave vials (5 mL and 20 mL) were from Biotage and were selected depending on the required solvent volume.

TiO_2_ NPs (AEROXIDE TiO_2_ P25) were purchased from Evonik Industries. The spherical NPs have an average radius of 10.5 nm (ref. 14) and an average surface area-to-volume ratio of 28%. The number of equivalents of NPs is defined as 0.28× the total number of TiO_2_ formula equivalents in the mass given, *i.e.* the effective surface concentration of TiO_2_.^11^ Similarly, when clarifying equivalents or mmol of functionalized NPs, it refers to the estimated amount of ligand or complex bound to the surface.

### Activation of commercial P25 TiO_2_ NPs

The commercial NPs were activated as previously described^11^ but the procedure was scaled up as follows. Commercial P25 TiO_2_ NPs (2.00 g) were dispersed by sonication for 15 min in dilute aqueous HNO_3_ (30 mL, 3 M). The mixture was then stirred for 30 min. The NPs were separated from the acid by centrifugation (10 min, 9000 rpm) and washed once with milliQ water (30 mL). The NPs were again dispersed in milliQ water (20 mL) through sonication for 10 min and the suspension was then stirred overnight. The NPs were separated by centrifugation (10 min, 9000 rpm) and washed with milliQ water (2 × 20 mL). The activated NPs (1.85 g) were stored in a sealed vial under N_2_ after drying over high vacuum.

### Preparation of (1)@TiO_2_ NPs

The functionalization was done as previously reported^11^ but the procedure was scaled up as follows. Ligand 1 (29.2 mg, 0.075 mmol, 1 eq.) and milliQ water (18 mL) were added to a microwave vial and dispersed through sonication for 1 min. Activated TiO_2_ NPs (660 mg, 30.8 TiO_2_ eq.) were added to the solution and the mixture was dispersed with sonication for 10 min. The microwave vial was sealed and the reaction mixture heated for 3 h at 130 °C in the microwave reactor. The (1)@TiO_2_ NPs were separated from the solvent by centrifugation (30 min, 9000 rpm) after cooling to room temperature. The white (1)@TiO_2_ NPs (674 mg) were stored in a sealed vial under N_2_ after drying under high vacuum. For NMR measurements, (1)@TiO_2_ NPs (5–10 mg) were dispersed in 500 μL D_2_O in an NMR tube.

### Attempted synthesis of [Rh_2_(μ-OAc)(1)_2_]Cl

Compound 1 (81.8 mg, 0.210 mmol, 2 eq.) was dispersed with NaCl (61.4 mg, 1.05 mmol, 10 eq.) through sonication for 10 min in ethanol (5 mL). [Rh_2_(μ-OAc)_4_(H_2_O)_2_] (50.2 mg, 0.105 mmol, 1 eq.) was added. Argon was bubbled through the suspension for 5 minutes while stirring. The suspension was stirred under argon at 80 °C for 18 h. The reaction mixture was allowed to cool down to room temperature and recrystallized from water to yield a dark solid. The product analysed as [Rh(1)_2_]Cl_3_ (71.9 mg, 0.0728 mmol, 69.3%). ^1^H NMR (500 MHz, D_2_O) *δ*/ppm: 9.31 (s, 4H, H^B3^), 8.84 (d, *J* = 8.1 Hz, 4H, H^A3^), 8.35 (ddd, 4H, *J* = 8.9, 6.8, 1.5 Hz, H^A4^), 8.22–8.15 (m, 4H, H^C2^), 8.11–8.05 (m, 4H, H^C3^), 7.82 (d, *J* = 5.6 Hz, 4H, H^A6^), 7.57 (ddd, *J* = 7.3, 5.6, 1.5 Hz, 4H, H^A5^). ^1^H NMR (500 MHz, TFA-*d*) *δ*/ppm: 9.27 (s, 4H, H^B3^), 8.90 (d, *J* = 8.0 Hz, 4H, H^A3^), 8.34–8.27 (m, 12H, H^A4+C2+C3^), 7.87 (d, *J* = 5.7 Hz, 4H, H^A6^), 7.59 (ddd, *J* = 7.5, 5.7, 1.3 Hz, 4H, H^A5^). ^13^C{^1^H} NMR (126 MHz, TFA-*d*) *δ*/ppm: 156.7 (C^B4^), 156.6 (C^A2^), 154.3, (C^B2^) 152.1 (C^A6^), 143.4 (C^A4^), 138.6 (C^C1^), 132.7 (C^C3^), 132.0 (C^C4^), 130.8 (C^A5^), 128.0 (C^C2^), 127.8 (C^A3^), 124.8 (C^B3^). MALDI: *m*/*z* 390.1 [(1) + H]^+^ (calc. 390.1), 492.0 [Rh(1)]^+^ (calc. 492.0), 681.0 [Rh(1) + CHCA]^+^ (calc. 681.0), 881.0 [Rh(1)_2_]^+^ (calc. 881.1), 903.0 [Rh(1)((1)–H)Na]^+^ (calc. 903.1).

### Synthesis of [Rh(1)_2_]Cl_3_

[Rh(1)_2_]Cl_3_ was prepared in an analogous manner to [Rh(4′-Phtpy)_2_][PF_6_]_3_.^15^ Ligand 1 (300 mg, 0.771 mmol, 2 eq.) was dispersed through sonication for 10 min in ethanol: water (1 : 1, 50 mL). RhCl_3_·3H_2_O (101 mg, 0.384 mmol, 1 eq.) was added. The solution was stirred at reflux (95 °C) for 5 h. The reaction mixture was allowed to cool down to room temperature and a precipitate formed which was filtered off and washed with water (3 × 10 mL), ethanol (3 × 10 mL) and Et_2_O (3 × 10 mL). [Rh(1)_2_]Cl_3_ (285 mg, 0.288 mmol, 75.0%) was collected as a black solid. ^1^H NMR (500 MHz, D_2_O) *δ*/ppm: 9.31 (s, 4H, H^B3^), 8.84 (d, *J* = 8.1 Hz, 4H, H^A3^), 8.35 (ddd, 4H, *J* = 8.1, 5.6, 1.4 Hz, H^A4^), 8.22–8.16 (m, 4H, H^C2^), 8.12–8.04 (m, 4H, H^C3^), 7.82 (d, *J* = 5.6 Hz, 4H, H^A6^), 7.57 (ddd, *J* = 7.5, 5.7, 1.4 Hz, 4H, H^A5^). ^1^H NMR (500 MHz, TFA-*d*) *δ*/ppm: 9.31 (s, 4H, H^B3^), 8.94 (d, *J* = 8.0 Hz, 4H, H^A3^), 8.41–8.29 (m, 12H, H^A4+C2+C3^), 8.01 (d, *J* = 5.5 Hz, 4H, H^A6^), 7.64 (dd, *J* = 8.0, 5.6, 1.4 Hz, 4H, H^A5^). ^13^C{^1^H} NMR (126 MHz, TFA-*d*) *δ*/ppm: 159.1 (C^B4^), 158.9 (C^A2^), 156.4, (C^B2^) 154.5 (C^A6^), 145.5 (C^A4^), 141.2 (C^C1^), 134.9 (d, *J*_PC_ = 11.4 Hz, C^C3^), 133.1 (d, *J*_PC_ = 194 Hz, C^C4^), 132.9 (C^A5^), 130.3 (d, *J*_PC_ = 16.4 Hz, C^C2^), 130.0 (C^A3^), 127.3 (C^B3^). MALDI: *m*/*z* 390.2 [(1) + H]^+^ (calc. 390.1), 492.0 [Rh(1)]^+^ (calc. 492.0), 527.0 [Rh(1) + Cl]^+^ (calc. 527.0), 681.1 [Rh(1) + CHCA]^+^ (calc. 681.0), 881.1 [Rh(1)_2_]^+^ (calc. 881.1), 903.2 [Rh(1)((1)–H) + Na]^+^ (calc. 903.1). Found C 48.85, H 3.54, N 7.99; C_42_H_38_Cl_3_N_6_O_9_P_2_Rh ([RhL_2_]Cl_3_·3H_2_O), requires C 48.41, H 3.68, N 8.07.

### Synthesis of [Rh(5)_2_][PF_6_]_3_

[Rh(5)_2_]Cl_3_ was prepared in an analogous manner to [Rh(4′-Phtpy)_2_][PF_6_]_3_.^15^ Compound 5 (150 mg, 0.337 mmol, 2 eq.) was dispersed through sonication for 10 min in ethanol : water (1 : 1, 35 mL). RhCl_3_·3H_2_O (44.2 mg, 0.168 mmol, 1 eq.) was added. The solution was stirred at reflux (95 °C) for 5 h. The reaction mixture was allowed to cool down to room temperature. The solution was filtered and NH_4_PF_6_ (82.2 mg. 0.504 mmol, 3 eq.) was added to the filtrate under stirring. A precipitate formed which was filtered off and washed with cold water (3 × 1 mL), cold EtOH (3 × 1 mL) and cold Et_2_O (3 × 1 mL). [Rh(5)_2_][PF_6_]_3_ (198 mg, 0.139 mmol, 82.7%) was collected as a pale pink solid. ^1^H NMR (500 MHz, CD_3_CN) *δ*/ppm: 9.14 (s, 4H, H^B3^), 8.77 (dd, *J* = 8.1, 1.4 Hz, 4H, H^A3^), 8.36–8.28 (m, 8H, H^A4+C2^), 8.21–8.15 (m, 4H, H^C3^), 7.75 (d, *J* = 5.6 Hz, 4H, H^A6^), 7.53 (ddd, *J* = 7.4, 5.6, 1.4 Hz, 4H, H^A5^). ^13^C{^1^H} NMR (126 MHz, CD_3_CN) *δ*/ppm: 157.7 (C^A2^), 156.4 (C^B4^), 154.9, (C^B2^) 153.9 (C^A6^), 143.8 (C^A4^), 140.0 (C^C1^), 133.7 (d, *J*_PC_ = 9.9 Hz, C^C3^), 133.5 (d, *J*_PC_ = 187 Hz, C^C1^), 131.2 (C^A5^), 129.6 (d, *J*_PC_ = 14.9 Hz, C^C2^), 128.6 (C^A3^), 126.3 (C^B3^), 63.4 (C^a^), 16.7 (C^b^). ^31^P{^1^H} NMR (200 MHz, CD_3_CN) *δ*/ppm: 16.06 (s, 2P, P(O)(OEt)_2_), −144.66 (heptet, *J*_PF_ = 707 Hz, 3P, [PF_6_]^−^). MALDI: *m*/*z*, 446.1 [(5) + H]^+^ (calc. 446.2), 548.0 [Rh(5)]^+^ (calc. 548.1), 737.1 [Rh(5) + CHCA]^+^ (calc. 737.1) 993.1 [Rh(5)_2_] (calc. 993.2), 1283.9 [Rh(5)_2_ + (PF_6_)_2_]^+^ (calc. 1283.2). ESI-MS: negative mode: *m*/*z* 145.06 [PF_6_]^−^ (calc. 144.96); positive mode: 331.26 [Rh(5)_2_]^3+^ (calc. 331.07), 569.27 [Rh(5)_2_ + (PF_6_)]^2+^ (calc. 569.09), 993.15 [Rh(5)_2_]^+^ (calc. 993.22), 1283.08 [Rh(5)_2_ + (PF_6_)_2_]^+^ (calc. 1283.14). HR ESI-MS: *m*/*z*, 331.0721 [Rh(5)_2_]^3+^ (calc. 331.0716), 496.1035 [Rh(5)((5)–H)]^2+^ (calc. 493.1038), 569.0895 [Rh(5)_2_ + (PF_6_)]^2+^ (calc. 569.0898), 1283.1423 [Rh(5)_2_ + (PF_6_)_2_]^+^ (calc. 1283.1444). UV-VIS (MeCN, 2.25 × 10^−5^ mol dm^−3^, *λ*/nm (*ε*/dm^3^ mol^−1^ cm^−1^): 243 (39 600), 284 (sh, 53 500), 295 (60 500), 329 (35 400), 342 (sh, 28 500), 361 (17 400).

### Crystallography

Single crystal data were collected on a Bruker APEX-II diffractometer (CuKα radiation) with data reduction, solution and refinement using the programs APEX,^16^ ShelXT,^17^ Olex2 (ref. 18) and ShelXL v. 2014/7.^19^ Structure analysis including the ORTEP representation, used CSD Mercury 2020.1.^20^ In the structure of [Rh(1)_2_][NO_3_]_3_·1.25[H_3_O][NO_3_]·2.75H_2_O, there are a large number of disordered water molecules, and one [NO_3_]^−^ position is partially occupied. For charge balance, 1.25H_2_O were treated as [H_3_O]^+^; this is reasonable because crystals were grown from concentrated HNO_3_. In [Rh(5)_2_][PF_6_]_3_·MeCN, due to high thermal motion, restraints were applied to two disordered ethoxy groups. Each phenylene ring was disordered and was modelled over two positions, 0.5 : 0.5 and 0.75 : 025 site occupancies, respectively. Each MeCN molecule was modelled with an occupancy of 0.5.

#### Crystal data for [Rh(1)_2_][NO_3_]_3_·1.25[H_3_O][NO_3_]·2.75H_2_O

C_42_H_41·25_N_10·25_O_22·75_P_2_Rh, *Mr* = 1218.45, brown block, triclinic, *P*1̄, *a* = 12.5277(7), *b* = 14.3560(8), *c* = 15.7458(9) Å, *α* = 91.845(2), *β* = 110.580(2), *γ* = 110.494(2)°, *V* = 2444.1(2) Å^3^, *T* = 150 K, *Z* = 2, μ(CuK_α_) = 4.300 mm^−1^. Total 23 158 reflections, 8879 unique (*R*_int_ = 0.0238). Refinement of 8743 reflections (712 parameters) with *I* > 2*σ*(*I*) converged at final *R*_1_ = 0.0486 (*R*_1_ all data = 0.0490), w*R*_2_ = 0.1365 (w*R*2 all data = 0.1371), gof = 1.085. CCDC 2040345.

#### Crystal data for [Rh(5)_2_][PF_6_]_3_·MeCN

C_52_H_51_F_18_N_7_O_6_P_5_Rh, *Mr* = 1469.76, colourless plate, triclinic, *P*1̄, *a* = 12.2477(7), *b* = 14.0636(8), *c* = 19.8182(11) Å, *α* = 73.903(4), *β* = 83.442(4), *γ* = 82.315(3)°, *V* = 3239.6(3) Å^3^, *T* = 150 K, *Z* = 2, μ(CuK_α_) = 4.223 mm^−1^. Total 37 630 reflections, 11 469 unique (*R*_int_ = 0.0431). Refinement of 9490 reflections (845 parameters) with *I* > 2*σ*(*I*) converged at final *R*_1_ = 0.1061 (*R*_1_ all data = 0.1189), w*R*_2_ = 0.2968 (w*R*2 all data = 0.3092), gof = 1.071. CCDC 2025772.

### Synthesis of Rh(1)_2_@TiO_2_ NPs

#### Method 1

(1)@TiO_2_ NPs (440 mg, 0.05 mmol, 2 eq.) were dispersed through sonication for 10 min in ethanol: water (1 : 1, 5 mL). RhCl_3_·3H_2_O (6.58 mg, 0.0250 mmol, 1 eq.) was added. The suspension was stirred at reflux (95 °C) for 5 h. The reaction mixture was allowed to cool down to room temperature. The Rh(1)_2_@TiO_2_ NPs were separated from the solvent by centrifugation (10 min, 9000 rpm) and then washed with water (2 × 8 mL) and ethanol (2 × 8 mL) and then dried under high vacuum yielding a light red powder (428 mg). Rh(1)_2_@TiO_2_ NPs (5–10 mg) was dispersed in 500 μL D_2_O in an NMR tube. MALDI spectrum of dried Rh(1)_2_@TiO_2_ NPs: *m*/*z* 390.1 [(1) + H]^+^ (calc. 390.1), 492.0 [Rh(1)]^+^ (calc. 492.0), 527.0 [Rh(1) + Cl]^+^ (calc. 527.0), 681.1 [Rh(1) + CHCA]^+^ (calc. 681.0), 881.1 [Rh(1)_2_]^+^ (calc. 881.1). Solid-state UV-VIS (*λ*/nm): 411, 608, 669.

#### Method 2

(1)@TiO_2_ NPs (440 mg, 0.05 mmol, 2 eq.) were dispersed with NaCl (14.6 mg, 0.25 mmol, 10 eq.) through sonication for 10 min in ethanol (5 mL). [Rh_2_(μ-OAc)_4_(H_2_O)_2_] (12 mg, 0.0251 mmol, 1 eq.) was added. Argon was bubbled through the suspension for 5 min while stirring. The suspension was stirred under argon at 80 °C for 18 h. The reaction mixture was allowed to cool down to room temperature. The Rh(1)_2_@TiO_2_ NPs were separated from the solvent by centrifugation (10 min, 9000 rpm) and then washed with water (2 × 8 mL) and ethanol (2 × 8 mL). The Rh(1)_2_@TiO_2_ NPs were dried under high vacuum yielding a light brown powder (430 mg). Rh(1)_2_@TiO_2_ NPs (5–10 mg) was dispersed in 500 μL D_2_O in an NMR tube. MALDI spectrum of dried (Rh(1)_2_@TiO_2_ NPs: *m*/*z* 390.1 [(1) + H]^+^ (calc. 390.1), 492.1 [Rh(1)]^+^ (calc. 492.0), 527.1 [Rh(1) + Cl]^+^ (calc. 527.0), 681.1 [Rh(1) + CHCA]^+^ (calc. 681.0). Solid-state UV-VIS (*λ*/nm): 444, 539, 609, 664.

### Oxidation of *rac*-(1*R*)-1-phenylethanol to acetophenone in water under air

Rh(1)_2_@TiO_2_ NPs (72.7 mg, 0.5 mol%) and milliQ water (1.67 mL) were added to a microwave vial. The contents were sonicated (1 min) and then aqueous NaOH (25 mM, 0.331 mL) and *rac*-(1*R*)-1-phenylethanol (0.1 mL, 0.827 mmol, 1 eq.) were added to the suspension. The vial was sealed and the reaction mixture was heated to 100 °C for 24 h. A small amount of the reaction mixture (*ca.* 50 μL) was removed by syringe and dispersed in 500 μL D_2_O in an NMR tube. ^1^H NMR spectra were measured to determine product : reactant ratios. The NMR spectroscopic data revealed that after 18 h, the ratio of product to reactant was 1.0 : 5.7 (14.9% product). The sampling procedure was repeated with 24 h and 38 h reaction time yielding a ratio of 1 : 3.3 (23.2% product, Table S1†) and 1 : 2.9 (25.7% product) respectively. The procedure yielded similar conversions independent of the synthetic route of the catalytic NPs.

### Recyclability of Rh(1)_2_@TiO_2_ NPs

Rh(1)_2_@TiO_2_ NPs (72.7 mg, 0.5 mol%) and milliQ water (1.67 mL) were added to a microwave vial. The suspension was sonicated (1 min) and then aqueous NaOH (25 mM, 0.331 mL) was added. The pH of the mixture was determined to be 7.8. *rac*-(1*R*)-1-Phenylethanol (0.1 mL, 0.827 mmol, 1 eq.) was added to the suspension after which the vial was sealed and the reaction mixture heated to 100 °C for 24 h. After cooling, the reaction mixture was washed with Et_2_O (6 × 3 mL). A small amount of the collected Et_2_O fractions (*ca.* 50 μL) was removed by syringe and the solvent was evaporated. The residue was dissolved in 500 μL D_2_O and added to an NMR tube. The ^1^H NMR spectrum was measured to determine product : reactant ratios. The pH of the reaction mixture was measured using a pH electrode and adjusted to the initial value of 7.8 by adding aqueous NaOH (25 mM). The vial was resealed and the reaction mixture was heated again to 100 °C for 24 h. The reaction was performed with this procedure 5 times in total. The product conversions were as follows: 20.3, 20.1, 18.3, 20.9 and 19.9%.

### Kinetics of oxidation

Rh(1)_2_@TiO_2_ NPs (145.4 mg, 0.5 mol%) and D_2_O (3.33 mL) were added into a microwave vial. The contents were sonicated (1 min) and then NaOH (25 mM in D_2_O, 0.667 mL) and *rac*-(1*R*)-1-phenylethanol (0.2 mL, 1.65 mmol, 1 eq.) were added to the suspension. The vial was sealed and the reaction mixture was heated to 100 °C for two weeks (*ca.* 300 h). At varying time intervals, a small amount of the reaction mixture (*ca.* 50 μL) was removed by syringe and dispersed in 500 μL D_2_O in an NMR tube. The ^1^H NMR spectrum was recorded to determine the product : reactant ratios at any point. Overall, data for 27 points were recorded over 12 days.

### Control experiment 1: catalyst influence on reaction rate

Activated NPs (72.7 mg) and milliQ water (1.67 mL) were added to a microwave vial. The contents were sonicated (1 min) and then aqueous NaOH (25 mM, 0.331 mL) and *rac*-(1*R*)-1-phenylethanol (0.1 mL, 0.827 mmol, 1 eq.) were added to the suspension. The vial was sealed and the reaction mixture was heated to 100 °C for 24 h. A small amount of the reaction mixture (*ca.* 50 μL) was removed by syringe and dispersed in 500 μL D_2_O in an NMR tube. The ^1^H NMR spectrum was recorded and revealed that no acetophenone had formed. Further control experiments were conducted using either unactivated commercial NPs, activated NPs, (1)@TiO_2_ NPs or [Rh_2_(μ-OAc)_4_(H_2_O)_2_]. The control experiment using (1)@TiO_2_ NPs and [Rh_2_(μ-OAc)_4_(H_2_O)_2_] showed 7% product conversion while all other experiments showed less than 1% product conversion after 24 hours (Table S1†).

### Control experiment 2: temperature influence on reaction rate

Rh(1)_2_@TiO_2_ NPs (72.7 mg, 0.5 mol%) and milliQ water (1.67 mL) were added to a microwave vial. The contents were sonicated (1 min) and then aqueous NaOH (25 mM, 0.331 mL) and *rac*-(1*R*)-1-phenylethanol (0.1 mL, 0.827 mmol, 1 eq.) were added to the suspension. The vial was sealed and the reaction mixture was left stirring at room temperature for 72 h. A small amount of the reaction mixture (*ca.* 50 μL) was removed by syringe and dispersed in 500 μL D_2_O in an NMR tube. The ^1^H NMR spectrum was recorded which revealed no acetophenone had formed (Table S1†).

### Control experiment 3: NaOH influence on functionalization stability

Rh(1)_2_@TiO_2_ NPs (72.7 mg, 0.5 mol%) and milliQ water (1.67 mL) were added to a microwave vial. The contents were sonicated (1 min) and then aqueous NaOH (25 mM, 0.331 mL) was added to the suspension. The vial was sealed and the reaction mixture was heated to 100 °C for 72 h. The supernatant solution was separated from the NPs by centrifugation (30 min, 17 500 rpm). The supernatant solution was filtered and added into a separate microwave vial and *rac*-(1*R*)-1-phenylethanol (0.1 mL, 0.827 mmol, 1 eq.) was added to the solution. The vial was sealed and the reaction mixture was heated to 100 °C for 24 hours. A small amount of the reaction mixture (*ca.* 500 μL) was removed by syringe and dispersed in 500 μL D_2_O in an NMR tube. The ^1^H NMR spectrum was recorded and revealed that no acetophenone had formed. The separated NPs were added together with milliQ water (1.67 mL) into a separate microwave vial. The contents were sonicated (1 min) and then aqueous NaOH (25 mM, 0.331 mL) *rac*-(1*R*)-1-phenylethanol (0.1 mL, 0.827 mmol, 1 eq.) were added to the suspension. The vial was sealed and the reaction mixture was heated to 100 °C for 24 hours. A small amount of the reaction mixture (*ca.* 50 μL) was removed by syringe and dispersed in 500 μL D_2_O in an NMR tube. The ^1^H NMR spectrum was recorded and showed acetophenone had been formed. This experiment was repeated without any base and yielded the same result. The reaction using the separated supernatant solution did not show acetophenone formation (<1%) while the reaction containing the separated NPs showed normal product conversion (19.9%).

### Control experiment 4: NaOH concentration influence on reaction rate

Rh(1)_2_@TiO_2_ NPs (72.7 mg, 0.5 mol%) and milliQ water (1.67 mL) were added to a microwave vial. The contents were sonicated (1 min) and then aqueous NaOH (25 mM, 0.331 mL) *rac*-(1*R*)-1-phenylethanol (0.1 mL, 0.827 mmol, 1 eq.) were added to the suspension. Simultaneously three other vials were prepared under the same conditions with different NaOH (0.331 mL, 50 mM, 0.25 M, 2.5 M) concentrations. The vials were sealed and the reaction mixtures were heated to 100 °C for 3 weeks. A small amount of each reaction mixture (*ca.* 50 μL) was removed from each vial by syringe and dispersed in 500 μL D_2_O in separate NMR tubes. The ^1^H NMR spectrum of each sample was recorded to determine the product : reactant ratios at any point. Overall, 11 data points were recorded over 24 days.

### Control experiment 5: oxidation using free rhodium complexes

[Rh(1)_2_]Cl_3_ (3.79 mg, *ca.* 0.5 mol%) and milliQ water (1.67 mL) were added to a microwave vial. The contents were sonicated (1 min) and then aqueous NaOH (25 mM, 0.331 mL) and *rac*-(1*R*)-1-phenylethanol (0.1 mL, 0.827 mmol, 1 eq.) were added to the suspension. The vial was sealed and the reaction mixture was left stirring at 100 °C for 24 h. A small amount of the reaction mixture (*ca.* 50 μL) was removed by syringe and dispersed in 500 μL D_2_O in an NMR tube. The NMR data revealed that after 24 h, the ratio of product to reactant was 1.0 : 5.3 (15.8% product, Table S1†). The procedure was repeated using [Rh(5)_2_][PF_6_]_3_ and showed a ratio of product to reactant of 1.0 : 4.2 (19.2% product, Table S1†).

### Control experiment 6: inert atmosphere and light influence on reaction rate

Four microwave vials were prepared to all of them Rh(1)_2_@TiO_2_ NPs (72.7 mg, 0.5 mol%) and milliQ water (1.67 mL) was added. The contents were sonicated (1 min) and then aqueous NaOH (25 mM, 0.331 mL) and *rac*-(1*R*)-1-phenylethanol (0.1 mL, 0.827 mmol, 1 eq.) were added to the suspensions. The suspensions in two vials were bubbled with Argon for 15 minutes. The vials were sealed and one under inert atmosphere and one under air were covered from light with alumina foil. Leading to vials having inert atmosphere, no light, inert atmosphere and no light, and a control with air not covered from light. The four vials were left stirring at 100 °C for 72 h. A small amount of each reaction mixture (*ca.* 50 μL) was removed by syringe and dispersed in 500 μL D_2_O in separate NMR tubes. The NMR data revealed that after 72 h, the ratio of product to reactant for the control vial was 1.0 : 2.4 (29.3% product, Table S1†) while the ratio of product to reactant when using inert gas was 1.0 : 5.0 (16.5% product, Table S1†). Performing the reaction without light had no effects on the product formation.

## Results and discussion

### Choice of anchoring ligand and substrate

2,2′:6′,2′′-Terpyridines (tpy) are well-established chelating ligands which undergo a conformational change from the equilibrium transoid-arrangement upon coordination to metal centres.^21^ The tpy metal-binding domain acts as a good σ-donor and a strong π-acceptor, making tpy ligands excellent candidates for the stabilization of low oxidation state metal centres. This leads to their applications in a wide range of homogeneous catalytic reactions involving metals such as Ni, Cu, Ru, Pd, Rh, Fe, Mg and Co.^22^

The functionalization of metal chalcogenide NPs with carboxylic and phosphonic acids is well-established.^23–32^ We have extended surface-modification strategies developed for dye-sensitized solar cells to nanoparticles and have illustrated that TiO_2_ NPs can be functionalized with bpy or tpy ligands bearing carboxylic or phosphonic acid anchoring units.^11^ We further demonstrated the preferential binding of phosphonic acids over carboxylic acids, and the ability of ligand-functionalized NPs to complex metal ions such as copper(i) and iron(ii) to form robust coordination-complex functionalized NPs.^11^

TiO_2_ NPs have benefits beyond being able to strongly bind anchoring ligands (carbocylic or phosphonic acids): they comprise earth abundant elements, are relatively cheap, non-toxic, thermodynamically stable and temperature resistant. TiO_2_ NPs can also be specifically prepared in a wide variety of sizes and shapes. This makes them a desirable choice for further investigation as a substrate for catalysis.

One potential problem with TiO_2_ NPs is that they can exhibit large amounts of surface-adsorbed water creating problems for catalytic processes in which it is crucial to avoid exposure to water and redox-related species such as dioxygen.^11,22^ It is therefore of significant interest to investigate catalytic systems that can tolerate both water and air.^12^ These “green” conditions are in any case desirable. The rhodium(iii) complexes selected for the present investigation are tolerant of both water and air.

### Attempted synthesis of [Rh_2_Cl_2_(μ-OAc)(1)_2_]Cl and synthesis of [Rh(1)_2_]Cl_3_

Wang and coworkers have reported the catalytic behaviour of the water–soluble complex [Rh_2_Cl_2_(μ-OAc)(tpy)_2_]Cl in the dehydrogenation and oxidation of alcohols in aqueous aerobic conditions to carboxylic acids or ketones.^12^ Our initial aim, was to prepare a TiO_2_ NP surface-bound version of this catalyst containing [Rh_2_Cl_2_(μ-OAc)(1)_2_]^+^. Before investigating the metallation of (1)@TiO_2_ NP, we decided to prepare [Rh_2_(μ-OAc)(1)_2_Cl_2_]Cl following the general procedure of Wang and coworkers, starting from [Rh_2_(μ-OAc)_4_(H_2_O)_2_]and 1 in the presence of NaCl in EtOH. A black product was isolated and the MALDI mass spectrum (Fig. S1†) showed peaks at *m*/*z* 492.0, 681.0, 881.1 and 903.0 corresponding to [Rh(1)]^+^, [Rh(1) + CHCA]^+^, [Rh(1)_2_]^+^ and [Rh(1)((1)–H) + Na]^+^. No peaks assigned to acetato species were observed in the mass spectrum and the ^1^H NMR spectrum (Fig. S2†) showed no signals arising from an acetate group.

A comparison of the ^1^H NMR spectrum of 1 with that of the black product suggested the formation of a homoleptic [Rh(1)_2_]^*n*+^ complex. The shift to lower frequency for the signal assigned to proton A6 (see Scheme 1) is consistent with this proton lying over the ring of a second tpy domain and the spectrum (which shows only one set of tpy signals) indicates the formation of a homoleptic bis(tpy) complex. We therefore concluded that the product was [Rh(1)_2_]Cl_3_. In order to confirm this proposal, we adapted the protocol described by Thomas and coworkers^15^ for the preparation of [Rh(4′-Phtpy)_2_][PF_6_]_3_ to prepare [Rh(1)_2_]Cl_3_ from RhCl_3_·3H_2_O and 1. The presence of chloride counter ion in the product was established by dissolving the compound in concentrated HNO_3_ and adding a drop of silver nitrate which lead to the precipitation of white silver chloride (Fig. S3†). The MALDI mass spectrum of [Rh(1)_2_]Cl_3_ (Fig. S4†) was similar to that described above (Fig. S2†) with peaks at *m*/*z* 492.0, 527.0, 681.1, 881.1 and 903.2 arising from [Rh(1)]^+^, [Rh(1)Cl]^+^, [Rh(1) + CHCA]^+^, [Rh(1)_2_]^+^ and [Rh(1)((1)–H) + Na]^+^. The ^1^H and ^13^C NMR spectra of [Rh(1)_2_]Cl_3_ (Fig. S5 and S6†) were assigned using 2D methods (Fig. S7–S9†) and were identical to those of the product from the attempted synthesis of [Rh_2_(μ-OAc)(1)_2_Cl_2_]Cl (Fig. S10–S13†). We speculate that the acidic phosphonic acid substituents on ligand 1 labilize the acetato ligands and prevent the isolation of [Rh_2_Cl_2_(μ-OAc)(1)_2_]^+^.

The compound [Rh(1)_2_]Cl_3_ formed during the synthesis was insoluble in most solvents and could only be dissolved in concentrated HNO_3_ or in water under very basic conditions. This pH dependent solubility suggested the formation of a zwitterionic species in basic conditions.

### Crystal structure of [Rh(1)_2_][NO_3_]_3_·1.25[H_3_O][NO_3_]·2.75H_2_O

**Fig. 1:**
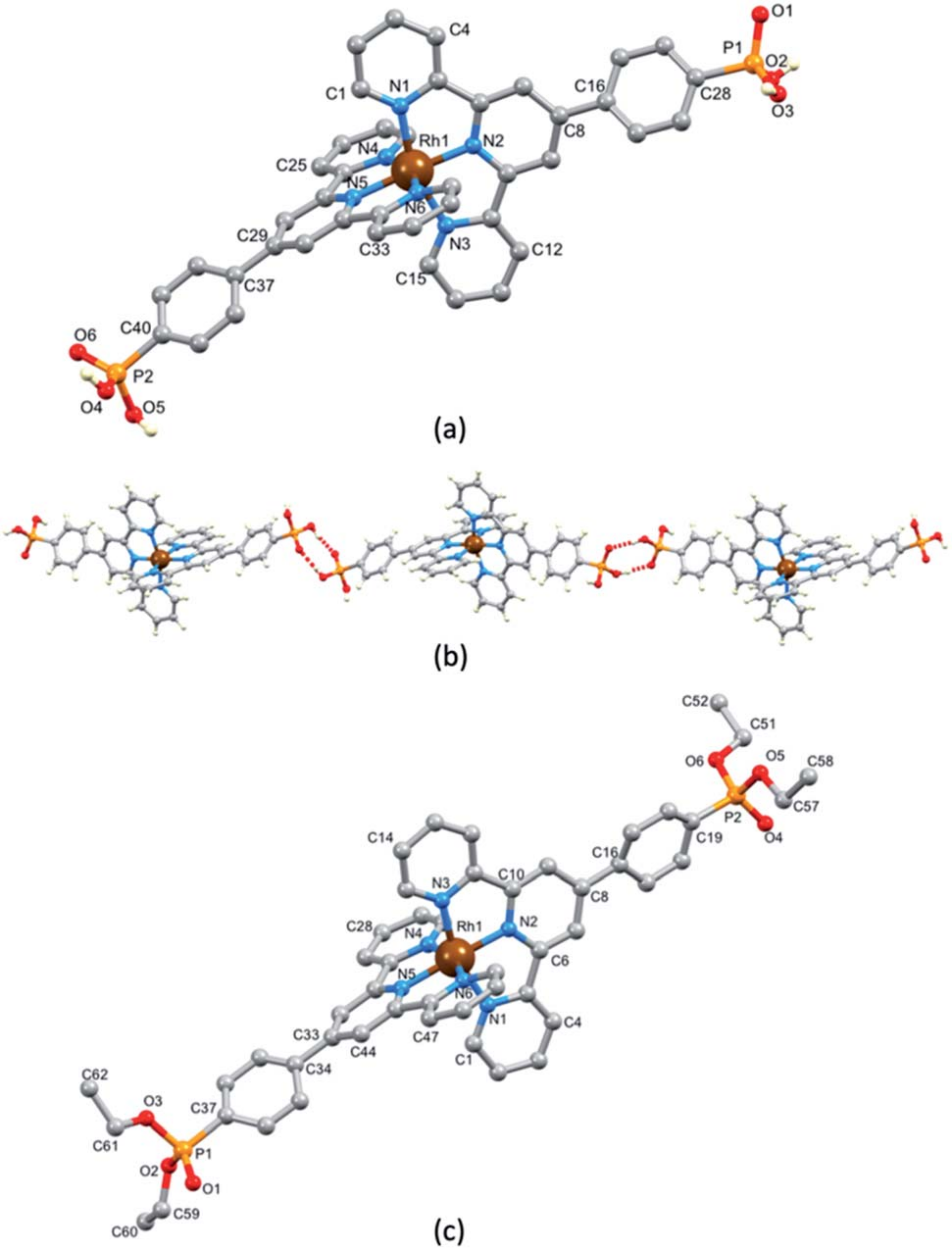
(a) The structure of the [Rh(1)_2_]^3+^ cation in the complex [Rh(1)_2_][NO_3_]_3_·1.25[H_3_O][NO_3_]·2.75H_2_O; H atoms except those in the phosphonic acid groups are omitted for clarity. Selected bond distances: Rh1–N1 = 2.047(3), Rh1–N2 = 1.962(3), Rh1–N3 = 2.055(3), Rh1–N4 = 2.055(3), Rh1–N5 = 1.964(3), Rh1–N6 = 2.043(3), P1–O1 = 1.501(3), P1–O2 = 1.544(3), P1–O3 = 1.552(3), P1–C28 = 1.786(4), P2–O4 = 1.550(3), P2–O5 = 1.546(4), P2–O6 = 1.489(3), P2–C40 = 1.800(4) Å. (b) Part of one hydrogen-bonded chain of [Rh(1)_2_]^3+^ cations. (c) The structure of the [Rh(5)_2_]^3+^ cation in [Rh(5)_2_][PF_6_]_3_·MeCN; H atoms are omitted for clarity. Selected bond parameters: Rh1–N1 = 2.040(6), Rh1–N2 = 1.964(6), Rh1–N3 = 2.046(7), Rh1–N4 = 2.047(7), Rh1–N5 = 1.984(6), Rh1–N6 = 2.050(7), P1–O1 = 1.471(10), P1–O2 = 1.540(15), P1–O3 – 1.605(14), P1–C37 = 1.803(11), P2–O4 = 1.453(8), P2–O5 = 1.707(15), P2–O6 = 1.634(13), P2–C19 = 1.787(9) Å.

Single crystals of [Rh(1)_2_][NO_3_]_3_·1.25[H_3_O][NO_3_]·2.75H_2_O grew from a solution of [Rh(1)_2_]Cl_3_ dissolved in a small amount of concentrated aqueous HNO_3_ which, after the addition of water, was left to stand at room temperature (*ca.* 22 °C) for 4 weeks. The complex crystallizes in the triclinic space group *P*1̄ and the single crystal structure confirmed the fully protonated phosphonic acid substituents. The crystal lattice contained a large number of disordered H_2_O molecules, as well as [H_3_O]^+^ and [NO_3_]^−^ in addition to the nitrate ions required to balance the charge on the [Rh(1)_2_]^3+^ cation. Because of the disorder, we focus only on the structure of the cation (Fig. 1a). An ORTEP representation of the cation is displayed in Fig. S21a.† The octahedral coordination environment with two chelating tpy domains is unexceptional with the [Rh(tpy)_2_} core closely resembling that observed in other [Rh(Xtpy)_2_]^3+^ cations (X = 4′-phenyl, 4′-(pyridin-4-yl), 4-ferrocenyl (DAHDAS,^15^ DAHDIA,^15^ DAHDEW,^15^ XIFTIS^34^). The Rh–N bond lengths are given in the caption to Fig. 1a, and the chelate N–Rh–N bond angles are in the range 79.84(13)–80.60(13)°. Each P atom is tetrahedrally sited and P–O and P–C bond lengths are given in the caption to Fig. 1a; the bond angles centred on P1 and P2 lie in the range 104.20(17)–114.96(18)°. The most dominant packing interaction involving the [Rh(1)_2_]^3+^ cations is hydrogen bonding between PO(OH)_2_ units leading to the assembly of 1D-chains (Fig. 1b). For the centrosymmetric hydrogen-bonded motifs, pertinent parameters are O1⋯O2^i^ = 2.485(5) Å, O1⋯H–O2^i^ = 1.68 Å, angle O1⋯H–O2^i^ = 159° (symmetry code i = −1 − *x*, −1 − *y*, 2 − *z*), O4⋯O6^ii^ = 2.534(5) Å, O4⋯HA–O6^ii^ = 1.70 Å, angle O4⋯HA–O6^ii^ = 171° (symmetry code ii = 1–*x*, 2–*y*, 1–*z*). Although the [Rh(1)_2_]^3+^ cations pack in the lattice with head-to-tail pairings of ligands on adjacent complexes, there are no significant π-stacking interactions between phenyl and tpy domains.

### Synthesis and characterization of [Rh(5)_2_][PF_6_]_3_

Because of initial uncertainties regarding the protonation state of the homoleptic complex obtained with 1, we prepared an analogous compound with a phosphonate ester. Compound 5 (Scheme 1) was prepared according to the literature^15^ and its reaction with RhCl_3_·3H_2_O followed by anion exchange using NH_4_PF_6_ yielded [Rh(5)_2_][PF_6_]_3_ as a pale pink solid in 82.7% yield. Mass spectrometric and ^1^H, ^13^C and ^31^P NMR spectroscopic data (see the Experimental section, Fig. S14–S16†) were consistent with the formation of the homoleptic complex. ^1^H and ^13^C NMR spectra were assigned using NOESY, HMQC and HMBC spectra (Fig. S17–S19†). The solution absorption spectrum (in MeCN) of [Rh(5)_2_][PF_6_]_3_ (Fig. S20†) exhibits intense bands below 370 nm which are assigned to ligand-centred π* ← π transitions similar to that reported for [Rh(tpy)_2_](PF_6_)_3_.^33^ The lack of absorptions in the visible region arising from metal-to-ligand charge transfer is consistent with the low-spin d^6^ configuration of the Rh(iii) centre. X-ray quality crystals of [Rh(5)_2_][PF_6_]_3_·MeCN were grown by vapour diffusion of Et_2_O into an MeCN solution and the structure of the [Rh(5)_2_]^3+^ cation is shown in Fig. 1c; an ORTEP representation is displayed in Fig. S21b.† Important bond lengths and angles are given in the caption to Fig. 1c. As anticipated, the Rh(iii) centre is octahedral, bound by two bis-chelating tpy domains. The structure is unexceptional but serves to confirm the formation of the homoleptic complex, and there are no noteworthy packing interactions.

### Assembly of a homoleptic rhodium(iii) complex on TiO_2_ NPs

Established procedures for TiO_2_ NP activation involve HNO_3_ (ref. 35) treatment and sonication to optimize surface functionalization and particle dispersion respectively.^36^ Commercial P25 TiO_2_ NPs were activated using aqueous HNO_3_ and then functionalized with ligand 1 (see the Experimental section). For the functionalization, an aqueous suspension of dispersed ligand 1 and activated NPs was heated to 130 °C for 3 h under microwave conditions. The (1)@TiO_2_ NPs were separated from the liquid phase by centrifugation. The functionalization procedure differs only from that previously described^11^ in that it was scaled up and the ratio TiO_2_ to 1 had to increased by 10% to ensure binding of all the ligand.

**Scheme 2:**
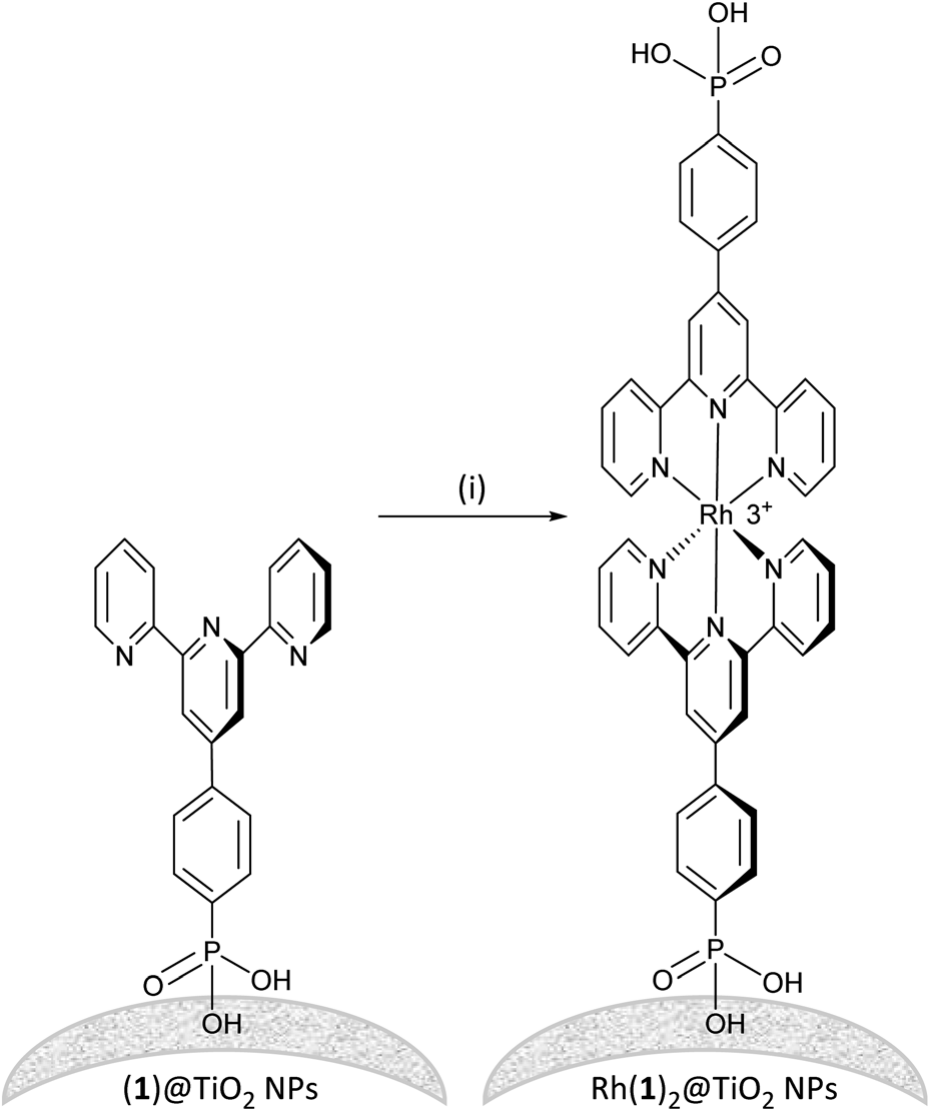
Assembly of [Rh(1)_2_]^3+^ on TiO_2_ NPs starting from NPs functionalized with 1. Conditions: (i) RhCl_3_, EtOH : H_2_O, 95 °C, 6 h or [Rh_2_(μ-OAc)_4_(H_2_O)_2_], NaCl, EtOH, 80 °C, 18 h. NP surfaces are rough and could potentially provide two binding sites for the rhodium metal complex or two NPs could come together to form the rhodium metal complex.

To form the NP-supported complex Rh(1)_2_@TiO_2_ (Scheme 2), (1)@TiO_2_ NPs were dispersed with RhCl_3_·3H_2_O in ethanol and water under air at 95 °C for 5 h. The NPs changed colour from white to pale red. The Rh(1)_2_@TiO_2_ NPs were separated from the solvent using centrifugation (see Experimental section, Method 1). The conjugate Rh(1)_2_@TiO_2_ was also prepared using [Rh_2_(μ-OAc)_4_(H_2_O)_2_] as a precursor (see Experimental, Method 2). (1)@TiO_2_ NPs were dispersed with NaCl and [Rh_2_(μ-OAc)_4_(H_2_O)_2_] in EtOH at 80 °C, and a colour change from pale blue to brown was observed during the reaction. The Rh(1)_2_@TiO_2_ NPs were separated using centrifugation. The overall charge associated with each {Rh(1)_2_} moiety in the Rh(1)_2_@TiO_2_ is uncertain as the protonation state and binding mode is typically not well defined.^37–39^ However, assuming that all 1 ligands are fully protonated, the Rh(1)_2_@TiO_2_ NPs should bear a +3 charge per surface-bound {Rh(1)_2_}.

The Rh(1)_2_@TiO_2_ NPs from both methods 1 and 2 were characterized using MALDI mass spectrometry and FTIR, ^1^H NMR and solid-state UV-VIS spectroscopies. FTIR spectra (Fig. S22†), solid-state absorption spectra (Fig. S23†), TGA measurements (Fig. S24 and S25†), MALDI mass spectra (Fig. S26 and S27†) and ^1^H NMR spectra (Fig. S28†) confirmed that both methods yielded similar Rh(1)_2_@TiO_2_ NPs. The characterization details are discussed further below.

### Characterization of Rh(1)_2_@TiO_2_ NPs

The MALDI mass spectrum of Rh(1)_2_@TiO_2_ NPs^+^ (Fig. S26 and S27†) showed peaks at *m*/*z* 492.1, 527.0 and 681.1 arising from [Rh(1)]^+^, [Rh(1) + Cl]^+^ and [Rh(1) + CHCA]^+^, respectively. Additionally, a peak at *m*/*z* 881.1 assigned to [Rh(1)_2_]^+^ was observed for Rh(1)_2_@TiO_2_ NPs prepared using method 1. The similarity between the mass spectrum of pristine [Rh(1)_2_]Cl_3_ and the mass spectra of Rh(1)_2_@TiO_2_ NPs is consistent with the assembly of the homoleptic [Rh(1)_2_]^3+^ complex on the NP surface using both [Rh_2_(μ-OAc)_4_(H_2_O)_2_] and RhCl_3_·3H_2_O as metal source.

**Fig. 2:**
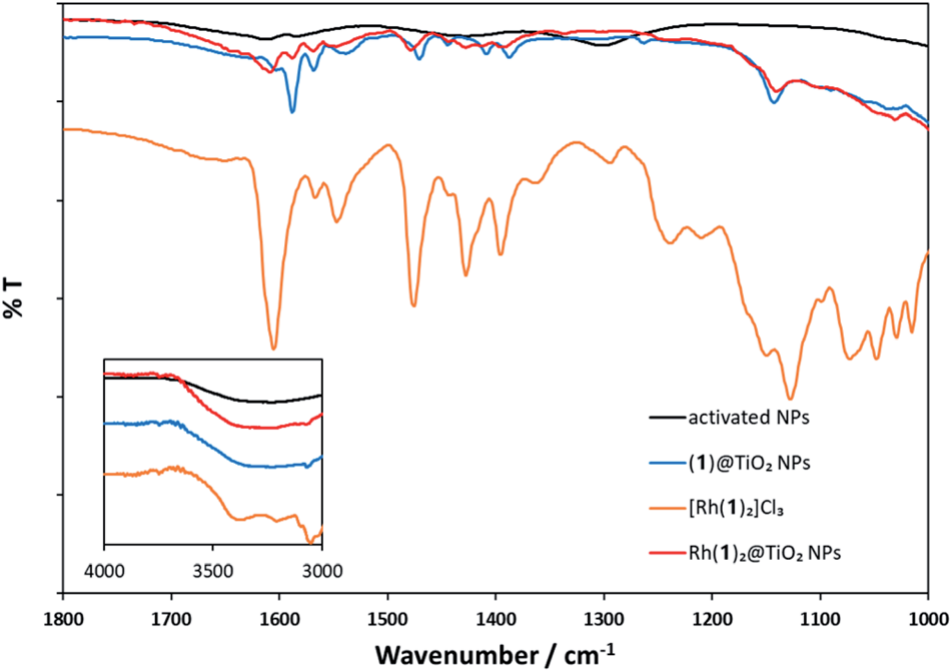
Solid-state FT-IR spectra of activated NPs (black), (1)@TiO_2_ NPs (blue), [Rh(1)_2_]Cl_3_ (orange), and Rh(1)_2_@TiO_2_ NPs (red).

Fig. 2 compares the FT-IR spectra of activated NPs, (1)@TiO_2_ NPs and Rh(1)_2_@TiO_2_ NPs together with the spectrum of the pristine compound [Rh(1)_2_]Cl_3_. The full spectra are presented in Fig. S29.† The activated NPs show weak absorption bands at 1613 and 1584 cm^−1^ while the (1)@TiO_2_ NPs exhibit bands at 1602, 1588, 1570, 1538, 1470, 1444, 1408 and 1387 cm^−1^. The isolated complex [Rh(1)_2_]Cl_3_ has absorption bands at 1605, 1565, 1549, 1477, 1429, 1395, 1367, 1297, 1242, 1213, 1152, 1127, 1072, 1048, 1029 and 1015 cm^−1^. Rh(1)_2_@TiO_2_ NPs shows bands at 1607, 1585, 1572, 1554, 1476, 1428, 1408, 1391 and 1136 cm^−1^ and their relative intensities and energies resemble most of the major peaks seen in (1)@TiO_2_ NPs and [Rh(1)_2_]Cl_3_.

Thus, the IR spectroscopic data provide evidence that (1)@TiO_2_ NPs has undergone a change upon treatment with either [Rh_2_(μ-OAc)_4_(H_2_O)_2_] or RhCl_3_·3H_2_O consistent with the formation of Rh(1)_2_@TiO_2_ NPs.

**Fig. 3:**
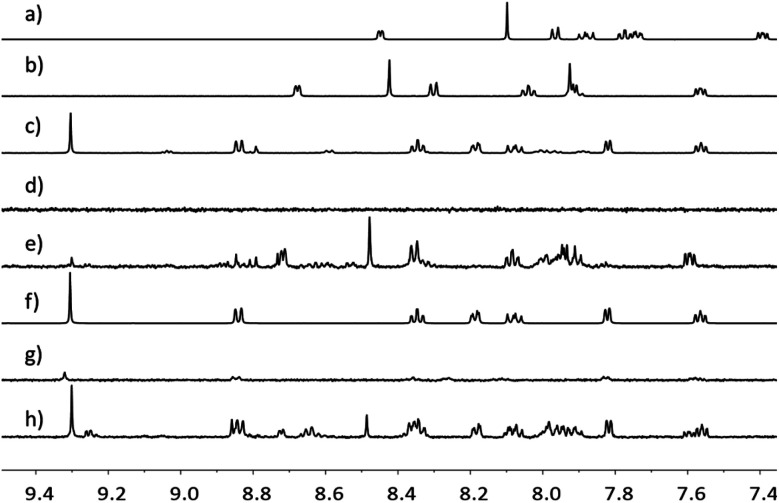
^1^H NMR spectra (500 MHz, D_2_O, 298 K) of 1 in D_2_O and NaOH (a), (1)@TiO_2_ in D_2_O and NaOH (b), [Rh(1)_2_]Cl_3_ made using [Rh_2_(μ-OAc)_4_(H_2_O)_2_] in D_2_O and NaOH (c), Rh(1)_2_@TiO_2_ NPs made using [Rh_2_(μ-OAc)_4_(H_2_O)_2_] in D_2_O (d), Rh(1)_2_@TiO_2_ NPs made using [Rh_2_(μ-OAc)_4_(H_2_O)_2_] in D_2_O and NaOH (e), [Rh(1)_2_]Cl_3_ made using RhCl_3_·3H_2_O in D_2_O and NaOH (f), Rh(1)_2_@TiO_2_ NPs made using RhCl_3_·3H_2_O in D_2_O (g), Rh(1)_2_@TiO_2_ NPs made using RhCl_3_·3H_2_O in D_2_O and NaOH (h). Chemical shifts in *δ*/ppm.

Further evidence for the NP functionalization came from ^1^H NMR spectroscopic data for Rh(1)_2_@TiO_2_ NPs dispersed in D_2_O. TiO_2_ NPs and their functionalized derivatives are insoluble in D_2_O, and signals associated with the surface-bound species are not observed under typical acquisition conditions. Any observed resonances can be attributed to the released of compounds from the surface. The ^1^H NMR spectrum of Rh(1)_2_@TiO_2_ NPs synthesized from [Rh_2_(μ-OAc)_4_(H_2_O)_2_] (Fig. 3d) showed no signals in the aromatic region. The only signals recorded arose from residual HOD and EtOH (Fig. S28†). A similar result was obtained for Rh(1)_2_@TiO_2_ NPs synthesized with RhCl_3_·3H_2_O (Fig. 3g). Under very basic conditions with an excess of NaOH, it is possible to partially defunctionalize the surface leading to the appearance of signals arising from free complex and ligand. This can be seen with the Rh(1)_2_@TiO_2_ NPs synthesized from [Rh_2_(μ-OAc)_4_(H_2_O)_2_] (Fig. 3e) or RhCl_3_·3H_2_O (Fig. 3h). The ^1^H NMR spectra of 1 (Fig. 3a), (1)@TiO_2_ and [Rh(1)_2_]Cl_3_ made with [Rh_2_(μ-OAc)_4_(H_2_O)_2_] (Fig. 3c) or RhCl_3_·3H_2_O (Fig. 3f) were also recorded under basic conditions. Using these spectra for comparison, we observed that the spectra of Rh(1)_2_@TiO_2_ NPs (Fig. 3e and h) contained signals arising from both 1 and [Rh(1)_2_]Cl_3_. This is not surprising considering that not every surface-bound ligand will bind a metal ion. However the similarity between the spectra of the pristine [Rh(1)_2_]Cl_3_ (Fig. 3c and f) and those of the defunctionalized NPs (Fig. 3e and h) provides strong evidence that the surface was partially functionalized with [Rh(1)_2_]^3+^ prior to treatment with base.

**Fig. 4:**
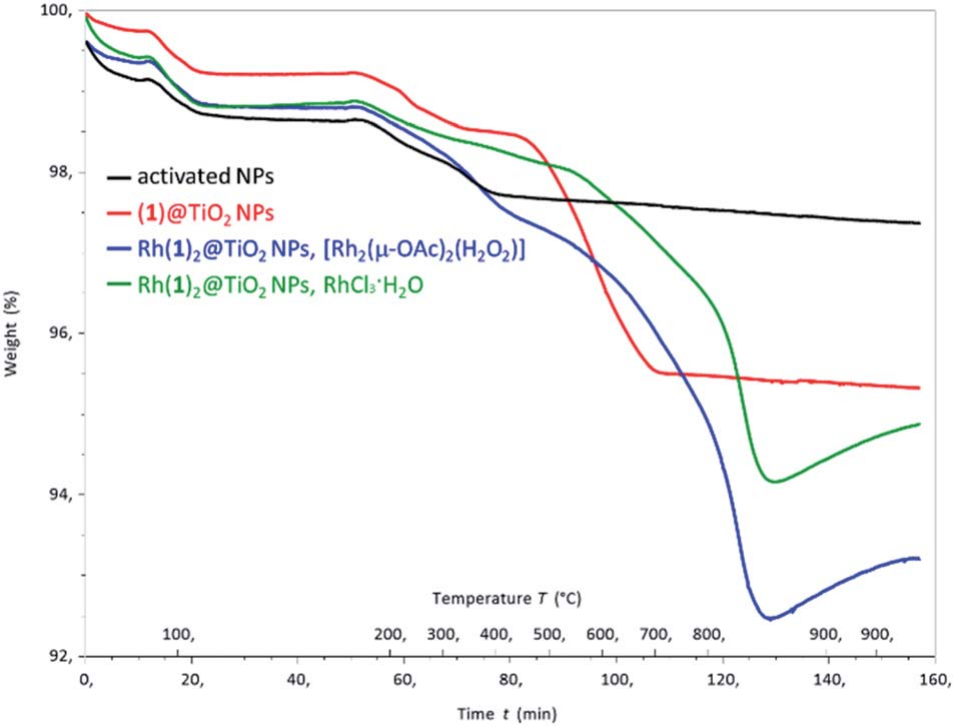
TGA curves for activated NPs (black), (1)@TiO_2_ NPs (red), Rh(1)_2_@TiO_2_ NPs made using [Rh_2_(μ-OAc)_4_(H_2_O)_2_] (blue) and for Rh(1)_2_@TiO_2_ NPs made using RhCl_3_·3H_2_O (green).

Thermogravimetric analysis (TGA) of activated NPs, (1)@TiO_2_ and Rh(1)_2_@TiO_2_ NPs was carried out, and the results are presented in Fig. 4. All samples show a weight loss of 1.5–2% in two steps (isotherm maxima <120 °C and <330 °C). The two steps can be attributed to the loss of physisorbed followed by chemisorbed water. The mass of the non-functionalized NPs undergoes no further significant change (Fig. S30†) upon being heated to 900 °C for 30 minutes. The (1)@TiO_2_ NPs and Rh(1)_2_@TiO_2_ NPs exhibit additional 3% and 4–5% weight losses above *ca.* 400 °C (Fig. S24 and S25†) ascribed to decomposition of the ligand. Additionally, Rh(1)_2_@TiO_2_ NPs show a weight increase occurring during the 30 minute 900 °C isotherm.

### NP supported homoleptic rhodium complex for alcohol oxidation

**Scheme 3:**
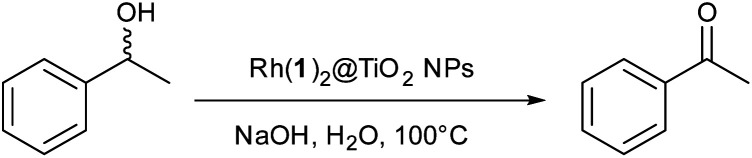
Secondary alcohol oxidation of *rac*-(1*R*)-1-phenylethanol to acetophenone.

Wang and coworkers^12^ reported the dehydrogenation and aerobic oxidation of alcohols under air to produce carboxylic acids or ketones with [Rh_2_Cl_2_(μ-OAc)(tpy)_2_]Cl as catalyst under aqueous conditions. We chose the reaction shown in Scheme 3 for a proof-of-principle investigation of the catalytic activity of Rh(1)_2_@TiO_2_ NPs.

**Fig. 5:**
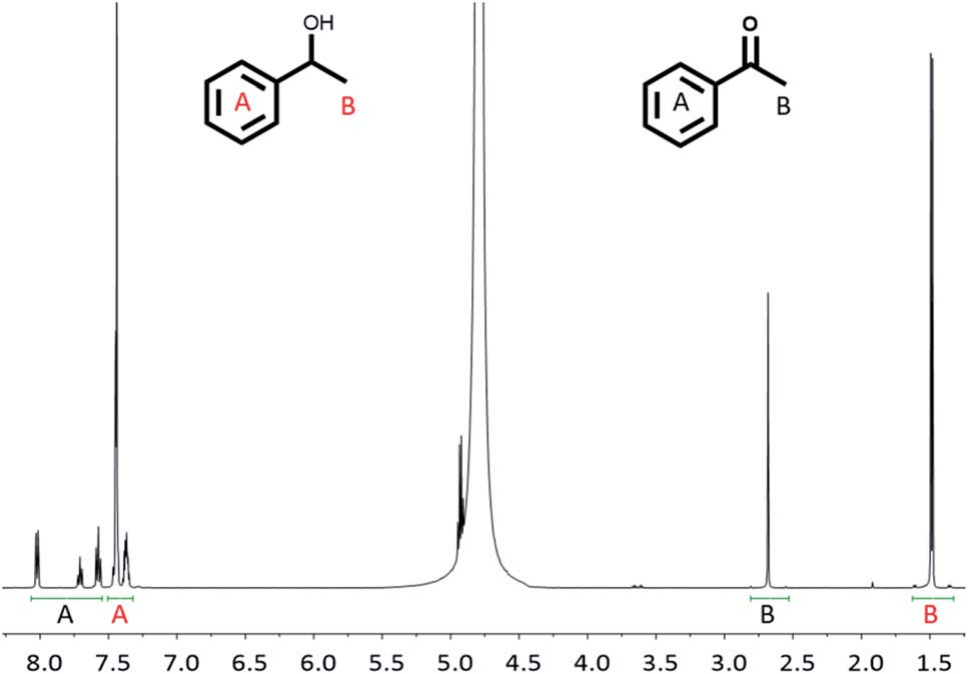
^1^H NMR spectrum (500 MHz, D_2_O, 298 K) of the reaction solution after alcohol oxidation (38 h) using Rh(1)_2_@TiO_2_ NPs as catalyst (see Experimental).

For the alcohol oxidation, Rh(1)_2_@TiO_2_ NPs were dispersed by sonication in milliQ water. Aqueous NaOH and *rac*-(1*R*)-1-phenylethanol were added to the suspension and the mixture was again dispersed by sonication. A control (Control experiment 3) was carried out to check that NaOH (at the concentrations used in the reactions) did not strip the catalyst from the surface. The reaction was performed under air at 100 °C for 18 h, 24 h and 38 h. The product : reactant ratio was measured using ^1^H NMR spectroscopy by removing a small amount of reaction solution and dispersing it in D_2_O (see Experimental section). Since the reaction shown in Scheme 3 involves one reactant forming one product, it was possible to reliably determine the reactant to product ratio by comparing the peak area of the aromatic protons and the methyl protons (Fig. 5).

Control experiments (see Experimental section) were performed to investigate several key factors during the reaction. Control experiment 1 compared Rh(1)_2_@TiO_2_ NPs with pristine commercial NPs or activated NPs were added to the reaction vial. After the reaction, ^1^H NMR spectroscopy revealed no acetophenone formation confirming that Rh(1)_2_@TiO_2_ NPs are the active catalyst. Control experiment 2 investigate the influence of temperature with the reaction being performed at room temperature (*ca.* 22 °C) instead of 100 °C. ^1^H NMR spectroscopy revealed <1% conversion to acetophenone, even after 72 h, at the lower temperature. Hence, elevated temperatures are important for product formation.

We also ensured that the reaction was catalysed by Rh(1)_2_@TiO_2_ NPs as opposed to [Rh(1)_2_]^3+^ that had been removed from the surface. Control experiment 3 investigated if defunctionalization could occur under the basic reaction conditions. Firstly, Rh(1)_2_@TiO_2_ NPs and NaOH were dispersed in milliQ water. The mixture was then heated to 100 °C for 72 h to simulate the reaction conditions. Next, the NPs were separated from the solution by centrifugation, and the NPs and the supernatant solution separated into two sample vials. The substrate *rac*-(1*R*)-1-phenylethanol was added to each vial (see Experimental section) and the reaction mixtures were then heated to 100 °C for 24 h. After the reaction, ^1^H NMR spectroscopy revealed no acetophenone had formed in the vial containing the supernatant solution whereas it was found in the vial containing the NPs. The results of Control 3 indicated that under the basic conditions used in the reaction, little or no defunctionalization of the NPs occurred.

**Fig. 6:**
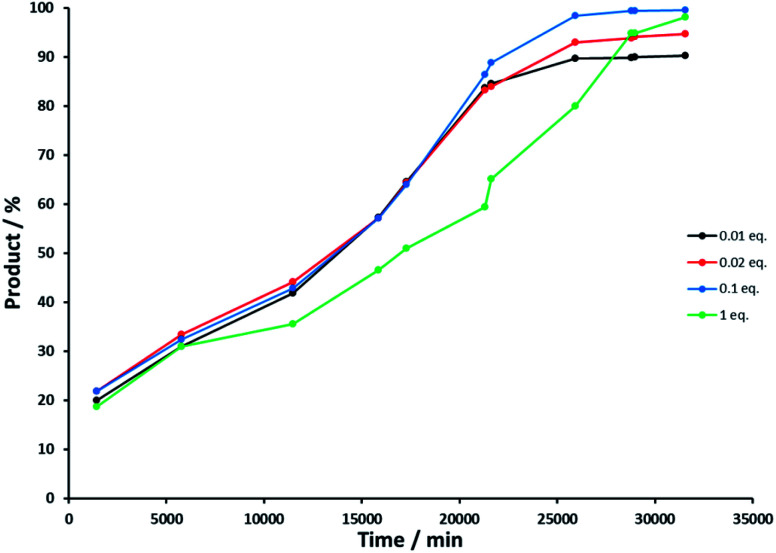
Product concentration in percent against time in minutes during alcohol oxidation with varing NaOH base concentration, NaOH equivalents compared to starting material: 0.01 (black), 0.02 (red), 0.1 (blue) and 1 (green).

Control experiment 4 investigated the influence of the concentration of the base concentration on the oxidation and allowed us to determine at what point defunctionalization occurred. Reactions were performed with NaOH – *rac*-(1*R*)-1-phenylethanol ratios of 0.01, 0.02, 0.1 and 1 (see Experimental section). Fig. 6 illustrates the reaction course of each experiment, and shows that the base concentration does not strongly influence the activity of Rh(1)_2_@TiO_2_ NPs with 0.01, 0.02 or 0.1 equivalents of NaOH. The catalytic activity of the Rh(1)_2_@TiO_2_ NPs is only affected strongly basic conditions (Fig. 6, green line).

Control experiment 5 was performed to compare the differences in catalytic activities of to [Rh(1)_2_]Cl_3_. We can make a number of general observations: (i) the attempt to prepare an immobilized dinuclear catalyst analogous to the established homogeneous species was unsuccessful (ii) the presence of the phosphonate functionality hinders the formation of the homogeneous dinuclear species (iii) both solution and surface chemistry leads to mononuclear complexes with 1 ligands (iv) the catalytic activity of homogeneous species depends to some extent upon the synthetic route used and (v) the catalytic activity of Rh(1)_2_@TiO_2_ NPs is generally similar to the homogeneous species.

Although [Rh(tpy)_2_]^*n*+^ species do not appear to have been used as photocatalysts, [Rh(bpy)_3_]^*n*+^ (*n* = 2 or 3) are well-established in multicomponent systems for photocatalytic reduction. We do not speculate in detail upon the mechanism of the photo-oxidation but it seems likely that the observed photooxidation product arises from the alcohol acting as a sacrificial reductant. We note that the yield of the oxidation product is somewhat reduced when the reaction is performed under argon (Table S1†). We have not observed dihydrogen production.^40–45^

### Kinetics of Rh(1)_2_@TiO_2_ NP catalysed alcohol oxidation

The kinetics of the alcohol oxidation by Rh(1)_2_@TiO_2_ NPs was investigated. The Rh(1)_2_@TiO_2_ NPs were dispersed in D_2_O, *rac*-(1*R*)-1-phenylethanol and aqueous NaOH were added and the mixture again dispersed by sonication. The alcohol oxidation was performed under air at 100 °C for two weeks. The product – reactant ratio was determined by measuring a small amount of reaction solution dispersed in D_2_O at certain time intervals with ^1^H NMR spectroscopy (see Experimental section).

**Fig. 7:**
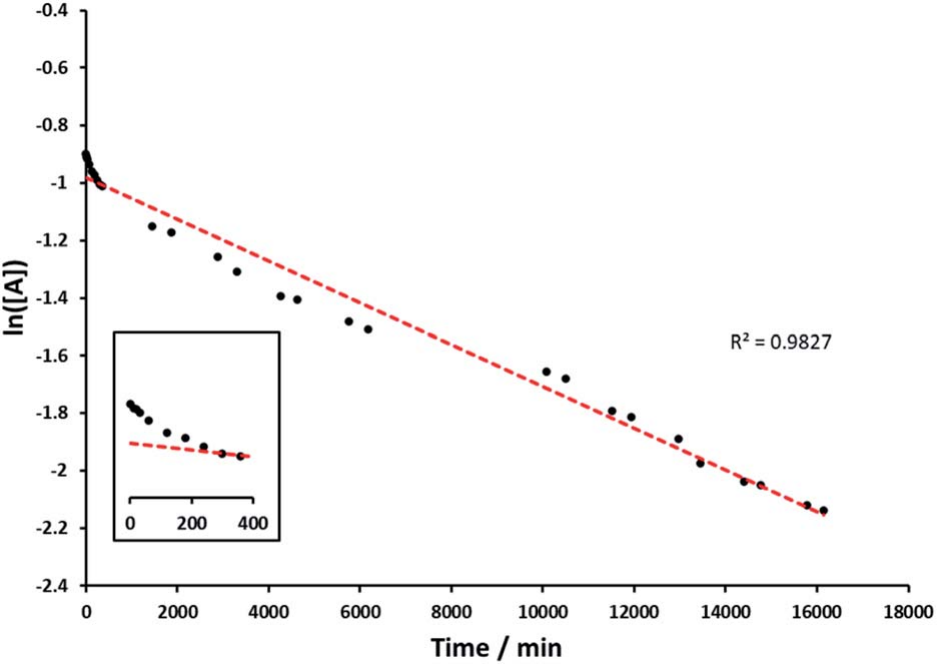
1st order reaction graph, natural logarithm of starting material concentration during kinetic measurements of *rac*-(1*R*)-1-phenylethanol oxidation (see Experimental) against time in minutes (black), linear trendline through datapoints (red), insert of first 400 minutes illustrates incubation time.

Analysis of the data indicated first order kinetics (Fig. 7). The data further suggested an incubation time during the first 6 h in which the reaction rate was slower. However overall *rac*-(1*R*)-1-phenylethanol was able to perform linearly over an extended period of time yielding over 71% product after the reaction.

### Recyclability of Rh(1)_2_@TiO_2_ NPs

The recyclability of the Rh(1)_2_@TiO_2_ NPs was studied. In contrast to the typical procedure described earlier, the pH was monitored using a pH electrode and was returned to pH 7.8 after each cycle. The conversion of each cycle was determined from the product and starting material extracted with Et_2_O from the reaction solution. This had the benefits of not removing any Rh(1)_2_@TiO_2_ NPs from the experiment with consequent distortion of the results from the next cycle. Five reaction cycles were measured under which the conversions stayed steady at a product to reactant ratio of 1.0 : 5 (20% product) after 24 hours reaction time.

## Conclusions

In this work we have built upon our previously established metal binding ligand functionalized TiO_2_ NPs and use their properties to form a surface bound homoleptic rhodium complex (Rh(1)_2_@TiO_2_ NPs). The functionalized NPs were investigated using TGA and ^1^H NMR and FT-IR spectroscopies. We further demonstrate a proof-of-principle investigation of their catalytic activity in an alcohol oxidation in water and under air. By tracking the product conversion over time with ^1^H NMR spectroscopy we were able to study the reaction kinetics and the recyclability of the catalyst. Rh(1)_2_@TiO_2_ NPs were further compared to their non-bound counterpart concluding that the NP-bound catalyst performs similarly if not slightly better. Rh(1)_2_@TiO_2_ NPs were able to withstand reactions at 100 °C for at least 24 days without showing decomposition or degradation. We further studied the resistance of the functionalization to higher base concentrations. Several other control experiments were performed to exclude influencing circumstances that could lead to unintentional product formation.

## Conflicts of interest

There are no conflicts to declare.

## Supplementary Material

RA-011-D0RA09319J-s001

RA-011-D0RA09319J-s002
