# Supplementary material for: Turning over on sticky balls: preparation and catalytic studies of surface-functionalized TiO2 nanoparticles
Source: RSC Adv. 2021 Jan 29;11(10):5537–47. doi: 10.1039/d0ra09319j (PMC8694772; doi:10.1039/d0ra09319j)
Supplement: RA-011-D0RA09319J-s001 [file RA-011-D0RA09319J-s001.pdf]

Electronic Supplementary Material (ESI) for RSC Advances.
This journal is © The Royal Society of Chemistry 2021

Supporting Information

Turning over on sticky balls: Preparation and catalytic studies of surface-functionalized TiO_2 nanoparticles

Sven A. Freimann,[a] Alessandro Prescimone,[a] Catherine E. Housecroft[a] and Edwin C. Constable*[a]

[a] Department of Chemistry, University of Basel, BPR 1096, Mattenstrasse 24a, CH-4058 Basel, Switzerland. email: edwin.constable@unibas.ch

Experimental Details of Instruments

^{1}H NMR, ^{13}C{^{1}H} NMR and ^{31}P{^{1}H} NMR spectra were measured at 298 K on a Bruker Avance III-500 NMR spectrometer. ^{1}H, ^{13}C and ^{31}P chemical shifts were referenced to residual solvent peaks with respect to δ(TMS) = 0 ppm for ^{1}H and ^{13}C{^{1}H} and δ(H_3PO_4 85% aqueous) = 0 ppm for ^{31}P{^{1}H}. A Gaussian fit to the diffusion peak intensity was done to determine the diffusion constant of the signal.

Reactions under microwave conditions were carried out in a Biotage Initiator 8 reactor. An Eppendorf Centrifuge 5415 R was used for 2 mL samples while a Hettich Centrifuge Universal 320 was used for 10 mL samples.

Solution absorption spectra were recorded on an Agilent Cary 5000 spectrophotometer and for solid-state absorption spectra, a Diffuse Reflectance Accessory was added to the spectrophotometer. For each solid-state absorption spectrum, a baseline correction was done with the respective nanoparticle precursor as the reference sample. FTIR spectra were recorded on a Perkin Elmer UATR Two spectrophotometer. Electrospray ionization (ESI) mass spectra and high resolution ESI MS were measured on a Shimadzu LCMS-2020 or a Bruker maXis 4G instrument, respectively. MALDI was measured on a Shimadzu MALDI-8020 with α-cyano-4-hydroxycinnamic acid (CHCA) solution as matrix for sample preparation.

Thermogravimetric analysis (TGA) was performed on a TGA5500 (TA Instruments) instrument under nitrogen. Initially, the temperature was held at 30 °C for 10 min before heating at a rate of 10 °C/min to 120 °C. This temperature was maintained for 30 min to remove all traces of water. Afterwards the sample was heated to 900 °C at a rate of 10 °C/min. After maintaining the temperature at 900 °C for 30 min, the sample was cooled to ambient temperature.

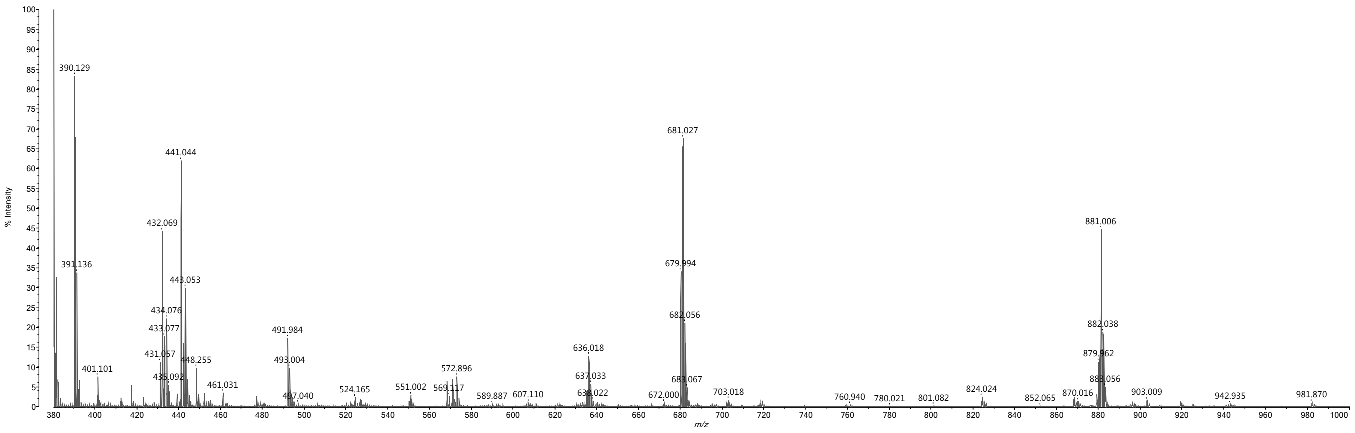

Fig. S1. MALDI mass spectrum (with CHCA matrix) of $[Rh(\mathbf{1})_2]Cl_3$ prepared from $[Rh_2(\mu\text{-}OAc)_4(H_2O)_2]$.

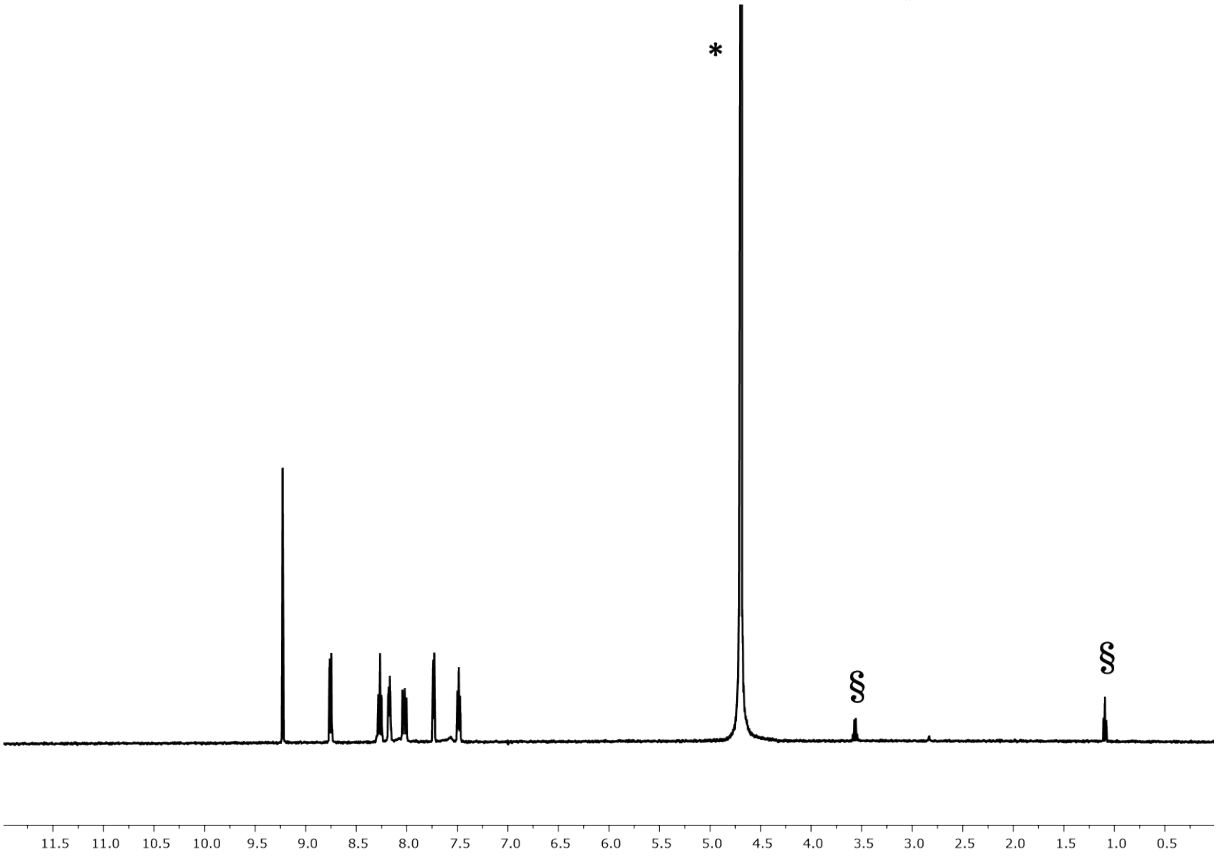

Fig. S2. ^{1}H NMR (500 MHz, D_2O, 298 K) spectrum of $[Rh(\mathbf{1})_2]Cl_3$ prepared from $[Rh_2(\mu\text{-}OAc)_4(H_2O)_2]$, * = HOD, § = residual EtOH. Chemical shifts in δ/ppm.

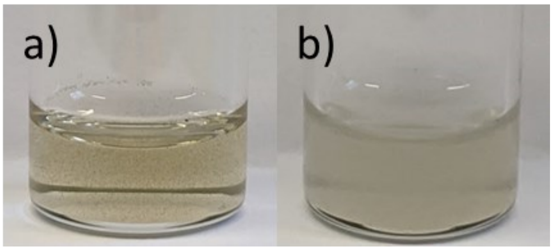

Fig. S3. a) $[Rh(\mathbf{1})_2]Cl_3$ dissolved in concentrated HNO_3 b) $[Rh(\mathbf{1})_2]Cl_3$ dissolved in concentrated HNO_3 after adding a drop of silver nitrate showing precipitation of AgCl.

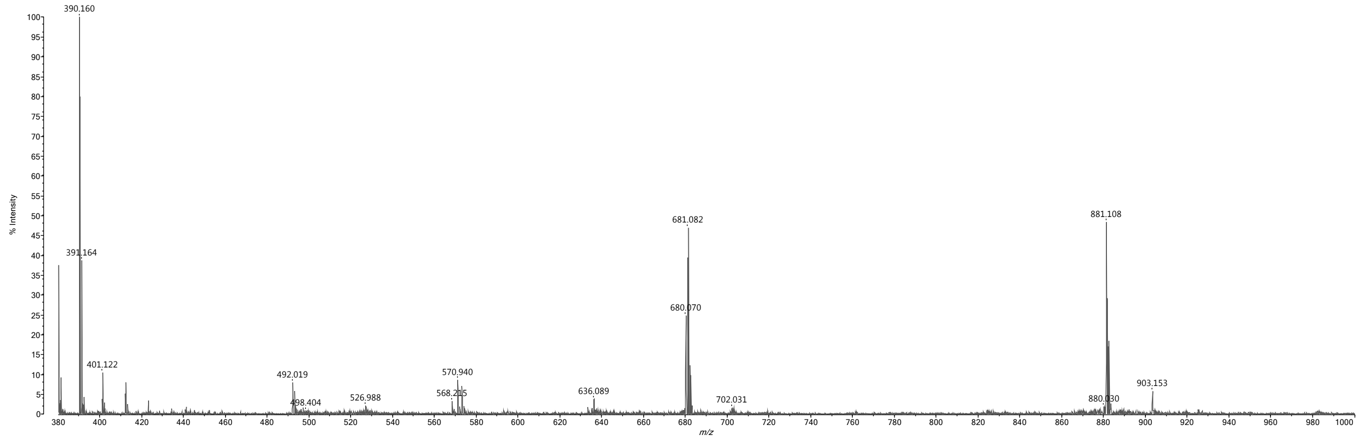

Fig. S4. MALDI mass spectrum (with CHCA matrix) of $[Rh(\mathbf{1})_2]Cl_3$ prepared from $RhCl_3.3H_2O$.

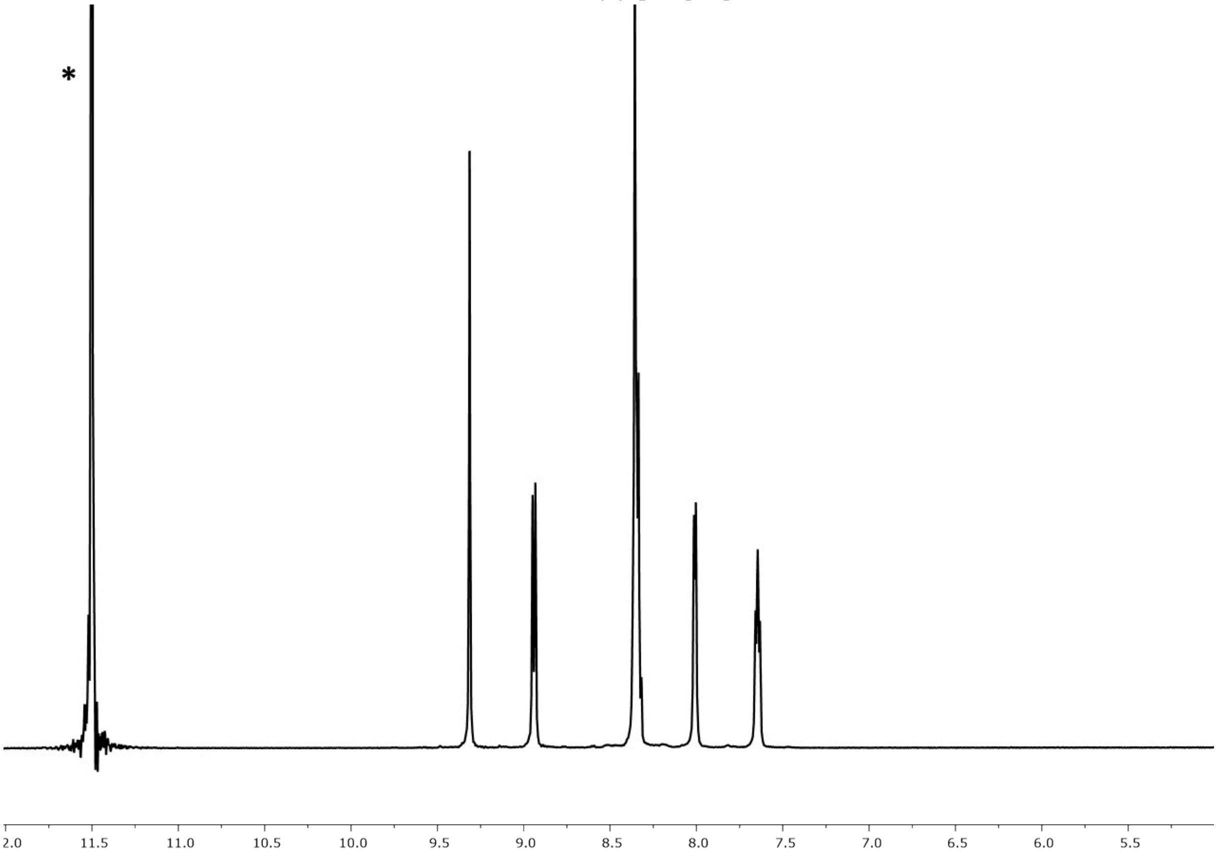

Fig. S5. ^{1}H NMR (500 MHz, TFA-d 298 K) spectrum of $[Rh(\mathbf{1})_2]Cl_3$ prepared from $RhCl_3.3H_2O$, * = HOD. Chemical shifts in δ/ppm.

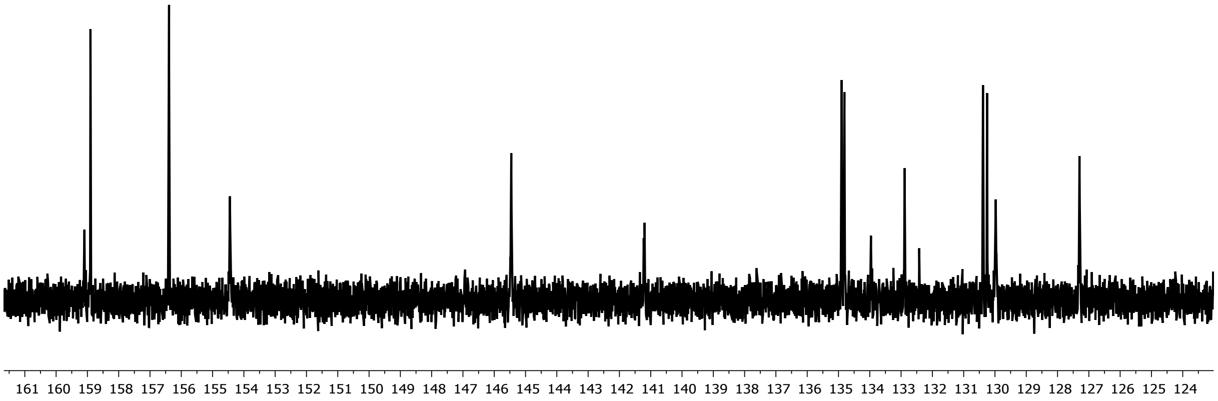

Fig. S6. $^{13}C\{^{1}H\}$ NMR (126 MHz, TFA-d 298 K) spectrum of $[Rh(\mathbf{1})_2]Cl_3$ prepared from $RhCl_3.3H_2O$. Chemical shifts in δ/ppm.

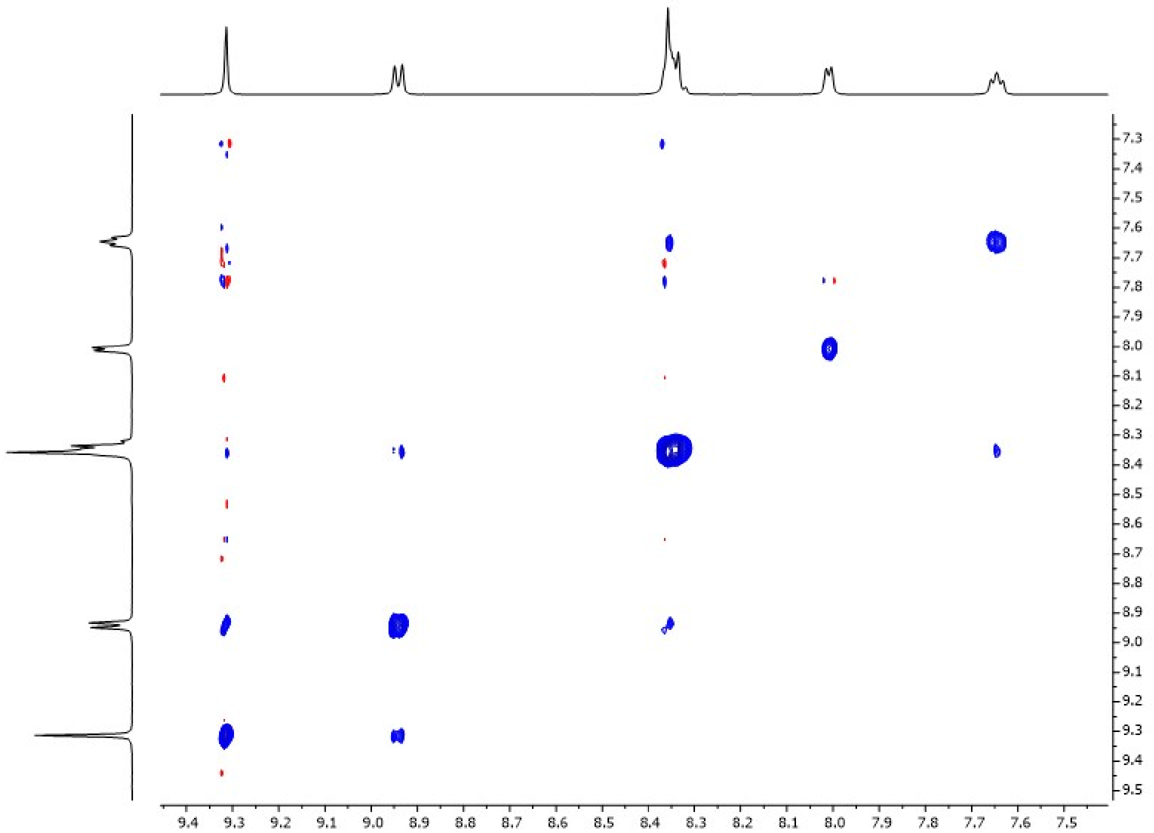

Fig. S7. NOESY (500 MHz, TFA-d 298 K) spectrum of [Rh(**1**)$_2$]Cl$_3$ prepared from RhCl$_3$.3H$_2$O. Chemical shifts in δ/ppm.

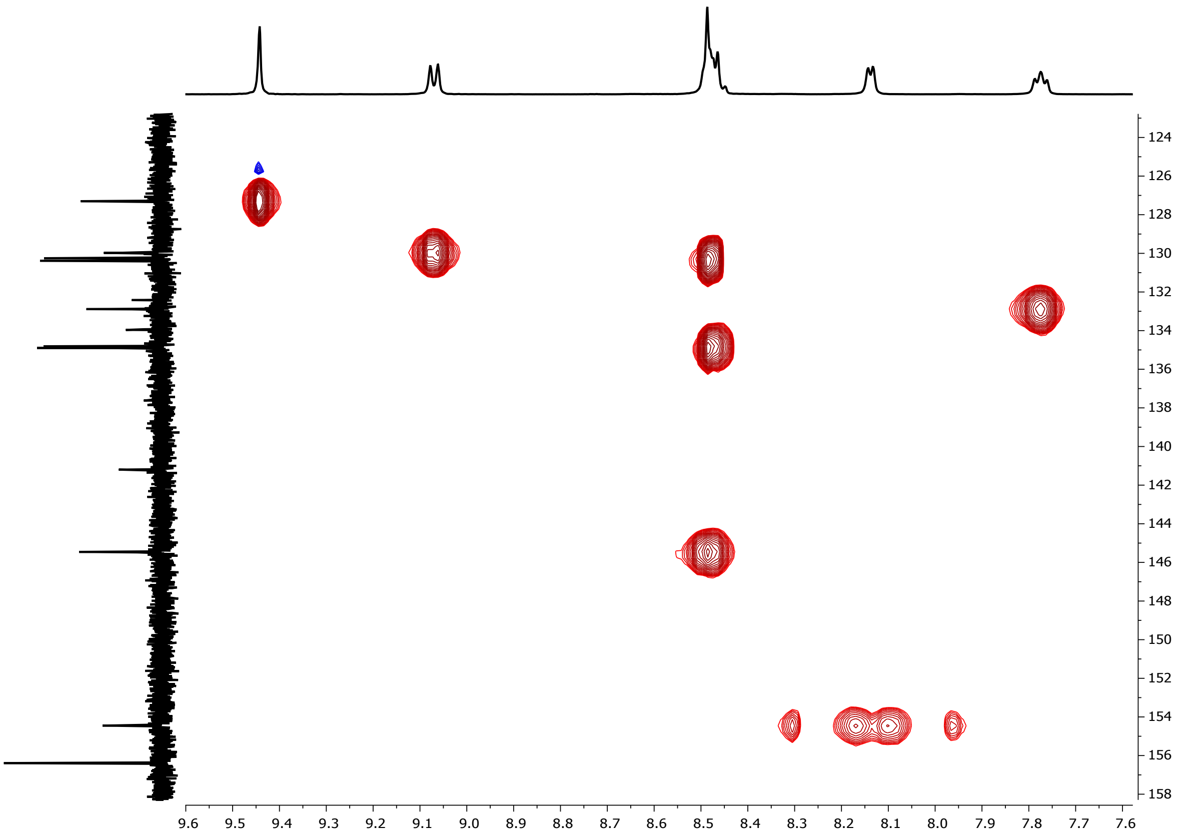

Fig. S8. HMQC (500 MHz ^{1}H, 126 MHz ^{13}C, TFA-d 298 K) spectrum of [Rh(**1**)$_2$]Cl$_3$ prepared from RhCl$_3$.3H$_2$O. Chemical shifts in δ/ppm.

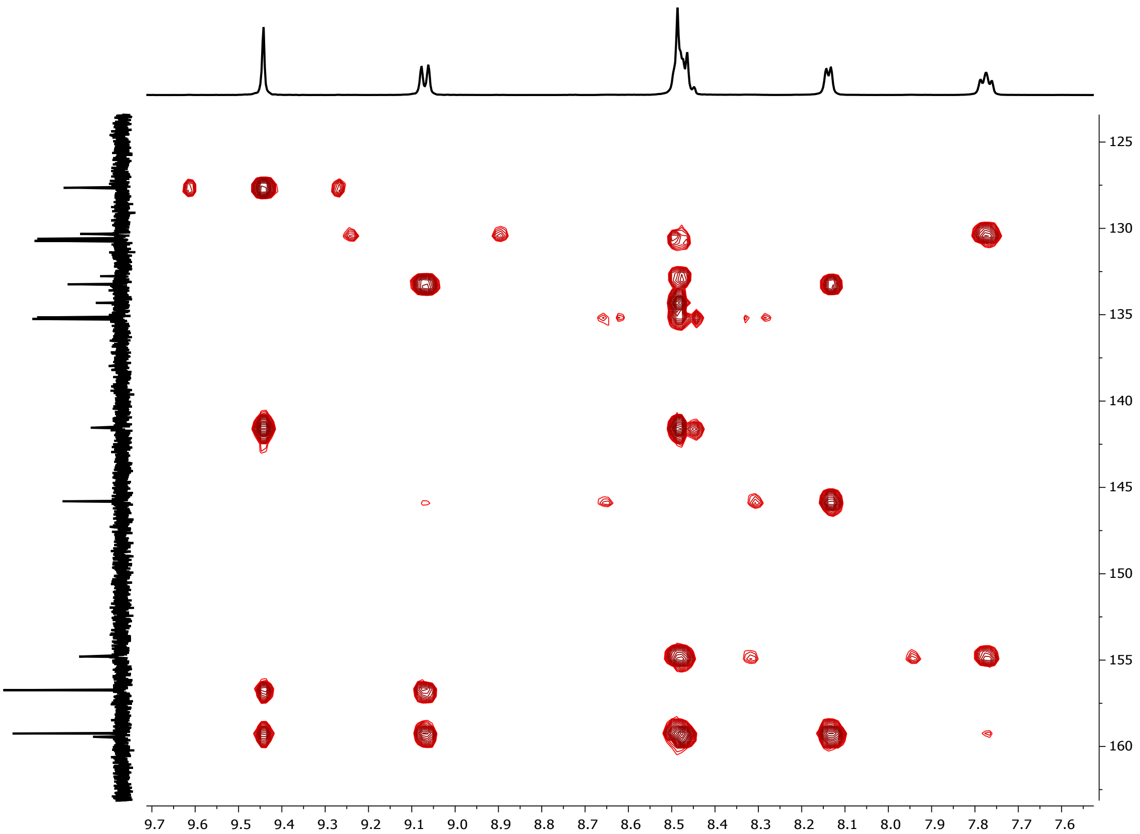

Fig. S9. HMBC (500 MHz ^{1}H, 126 MHz ^{13}C, TFA-d 298 K) spectrum of $[Rh(\mathbf{1})_2]Cl_3$ prepared from $RhCl_3.3H_2O$. Chemical shifts in δ/ppm.

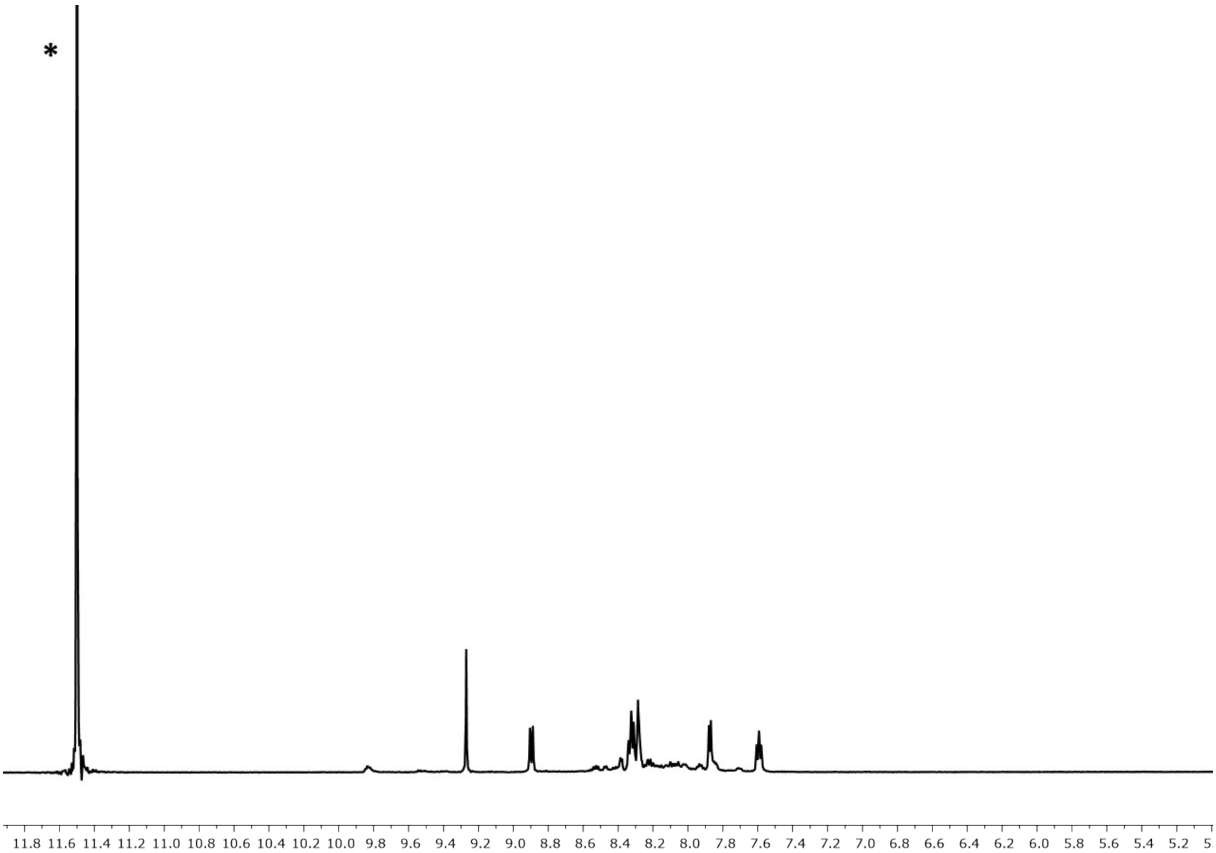

Fig. S10. ^{1}H NMR (500 MHz, TFA-d, 298 K) spectrum of $[Rh(\mathbf{1})_2]Cl_3$ prepared from $[Rh_2(\mu\text{-}OAc)_4(H_2O)_2]$, * = CF_3CO_2H. Chemical shifts in δ/ppm.

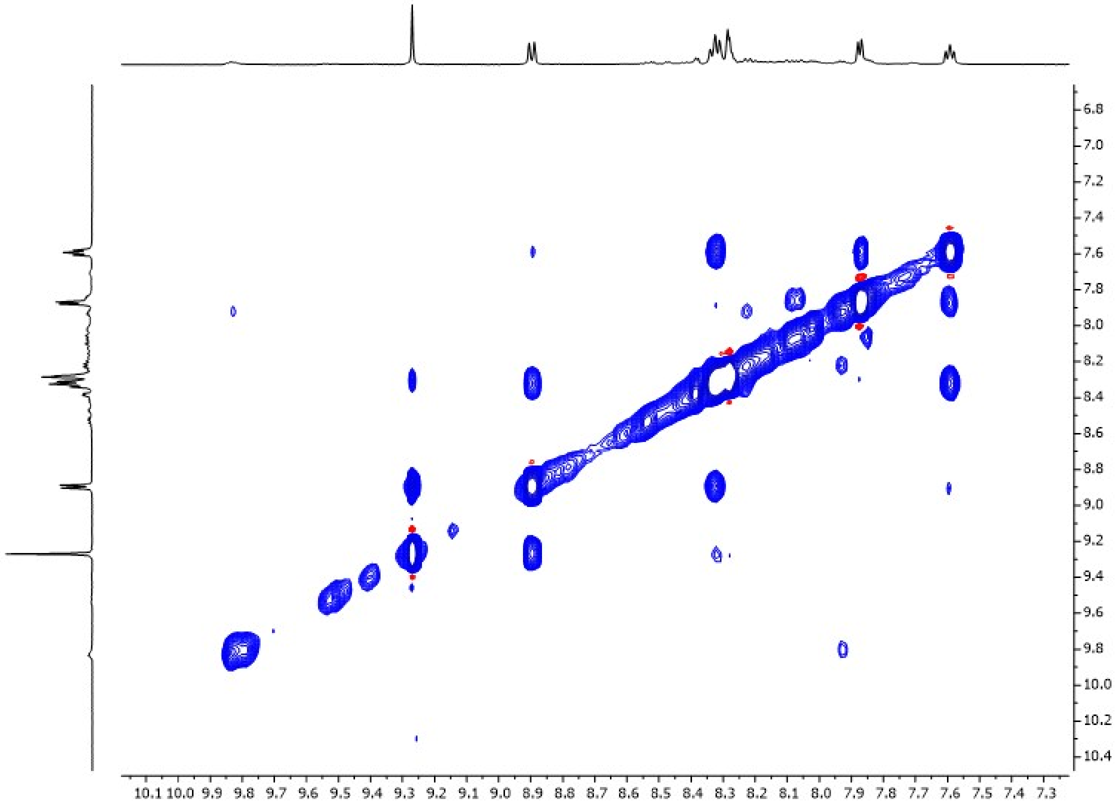

Fig. S11. NOESY (500 MHz, TFA-d 298 K) spectrum of $[Rh(\mathbf{1})_2]Cl_3$ prepared from $[Rh_2(\mu\text{-}OAc)_4(H_2O)_2]$. Chemical shifts in δ/ppm.

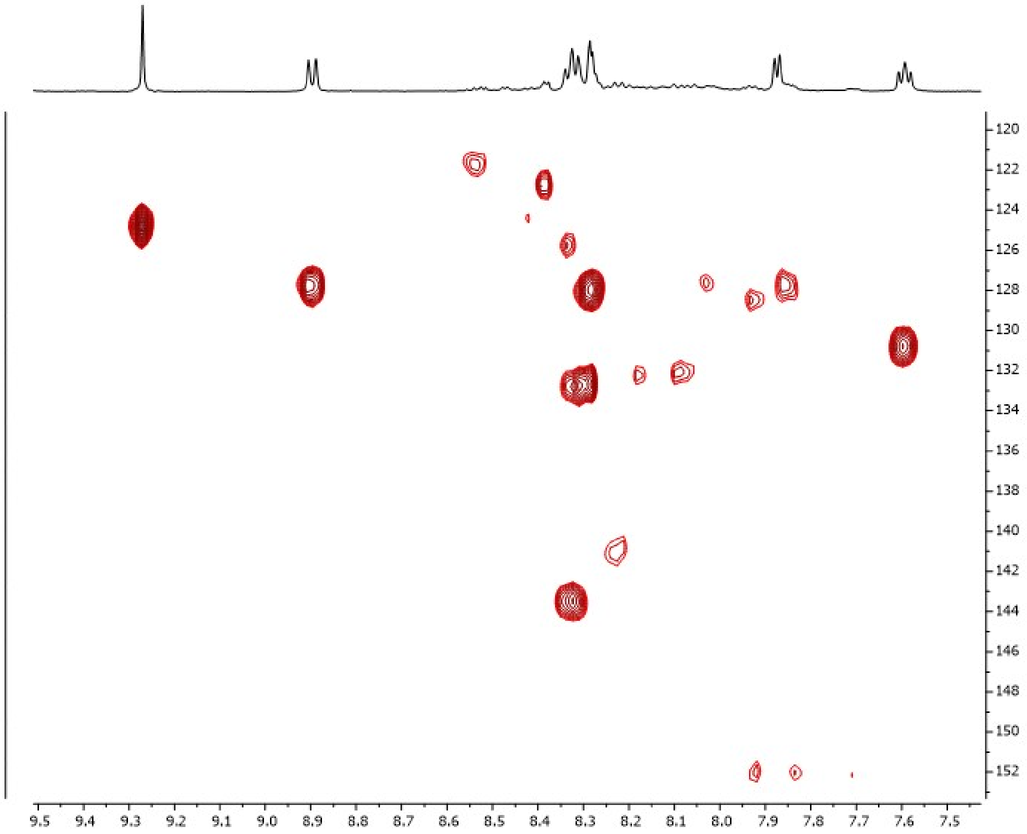

Fig. S12. HMQC (500 MHz ^{1}H, 126 MHz ^{13}C, TFA-d 298 K) spectrum of $[Rh(\mathbf{1})_2]Cl_3$ prepared from $[Rh_2(\mu\text{-}OAc)_4(H_2O)_2]$. Chemical shifts in δ/ppm.

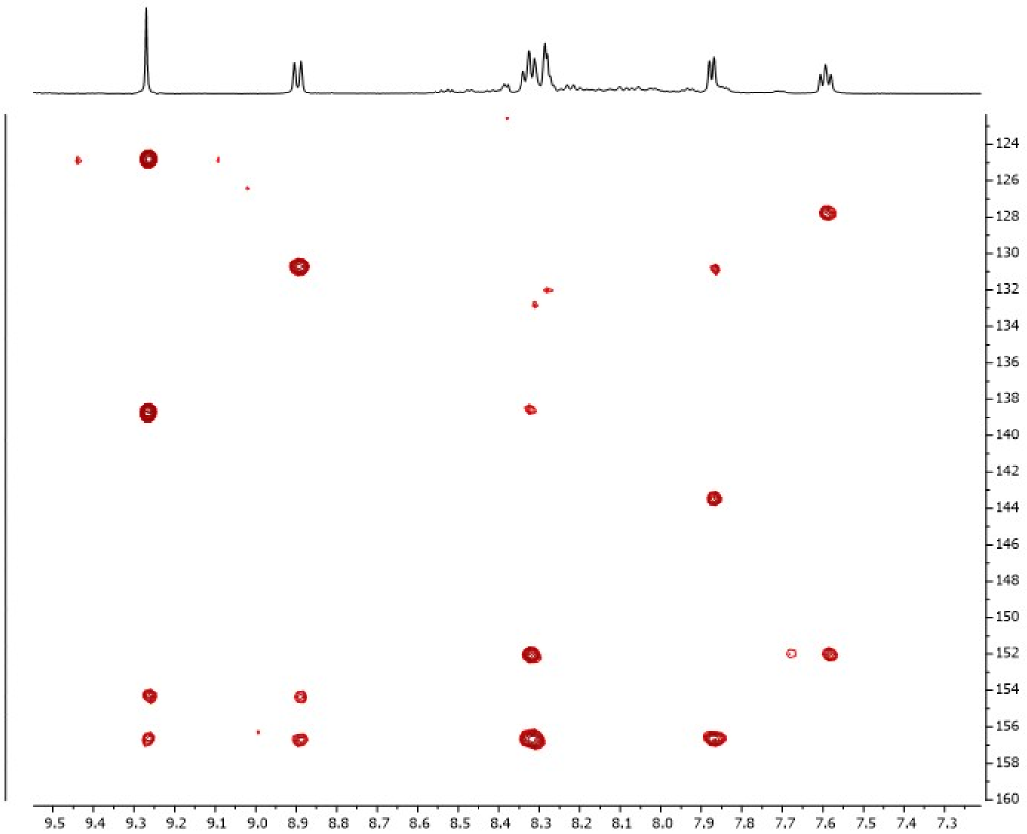

Fig. S13. HMBC (500 MHz ^{1}H, 126 MHz ^{13}C, TFA-d 298 K) spectrum of [Rh(**1**)$_2$]Cl_3 prepared from [Rh_2(μ-OAc)$_4$(H_2O)$_2$]. Chemical shifts in δ/ppm.

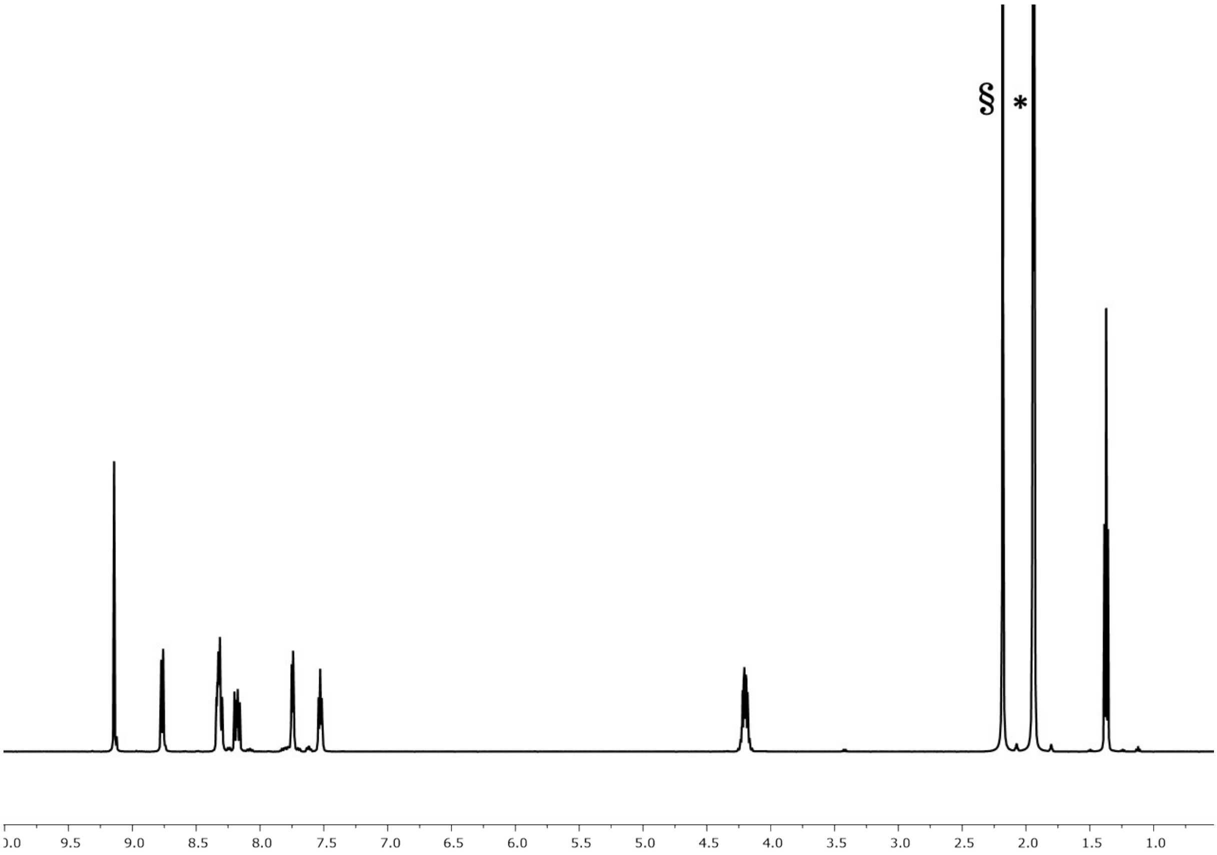

Fig. S14. ^{1}H NMR (500 MHz, CD_3CN, 298 K) spectrum of [Rh(**5**)$_2$][PF_6]$_3$, * = CHD_2CN, § = HOD. Chemical shifts in δ/ppm.

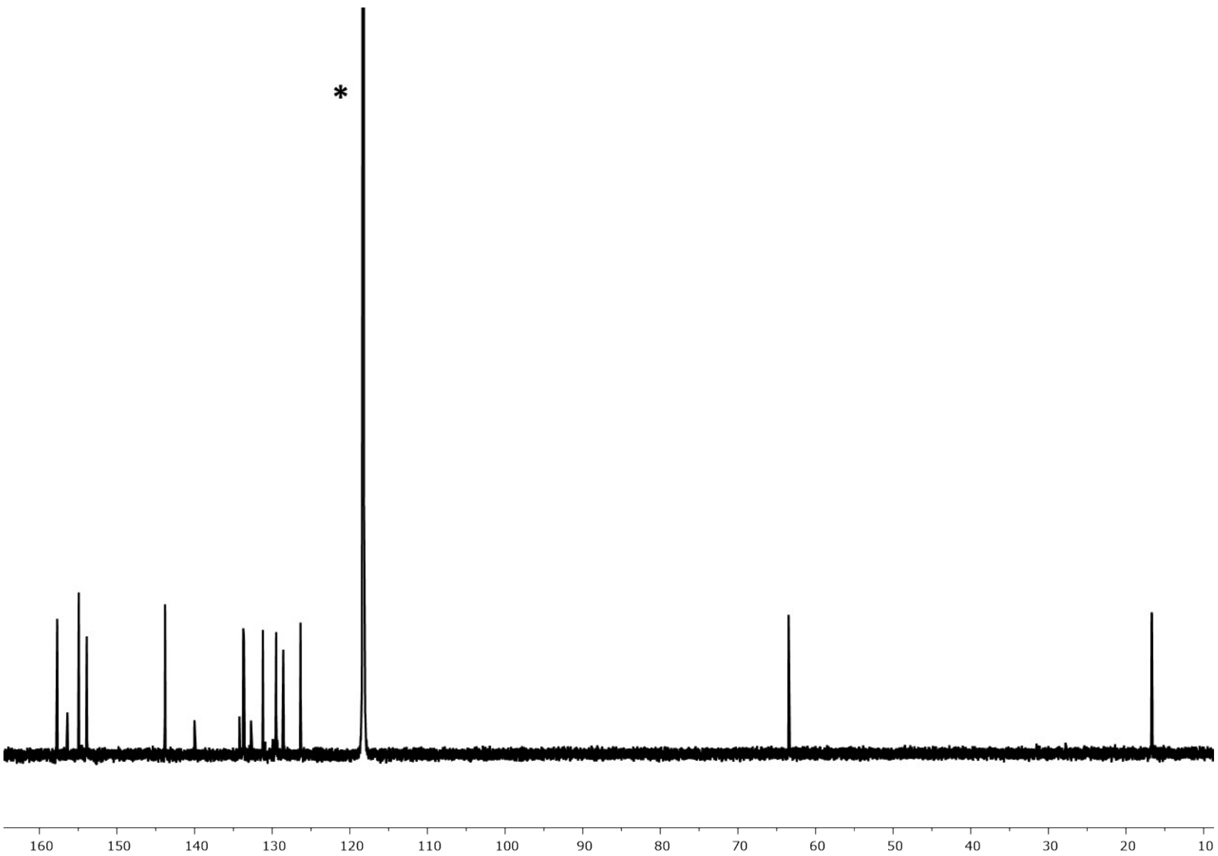

Fig. S15. $^{13}C\{^{1}H\}$ NMR (126 MHz, CD_3CN, 298 K) spectrum of $[Rh(\mathbf{5})_2][PF_6]_3$, * = CHD_2CN. Chemical shifts in δ/ppm.

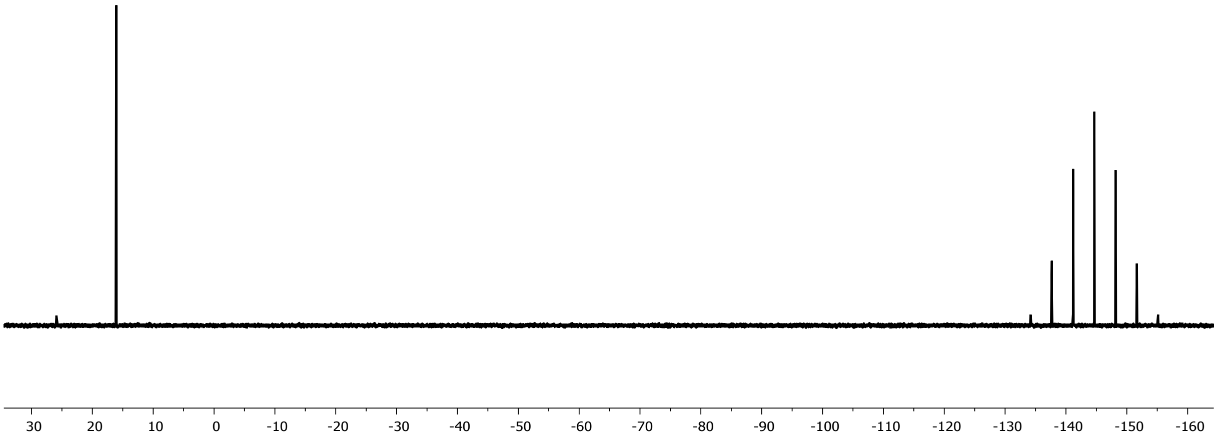

Fig. S16. $^{31}P\{^{1}H\}$ NMR (202 MHz, CD_3CN, 298 K) spectrum of $[Rh(\mathbf{5})_2][PF_6]_3$. Chemical shifts in δ/ppm.

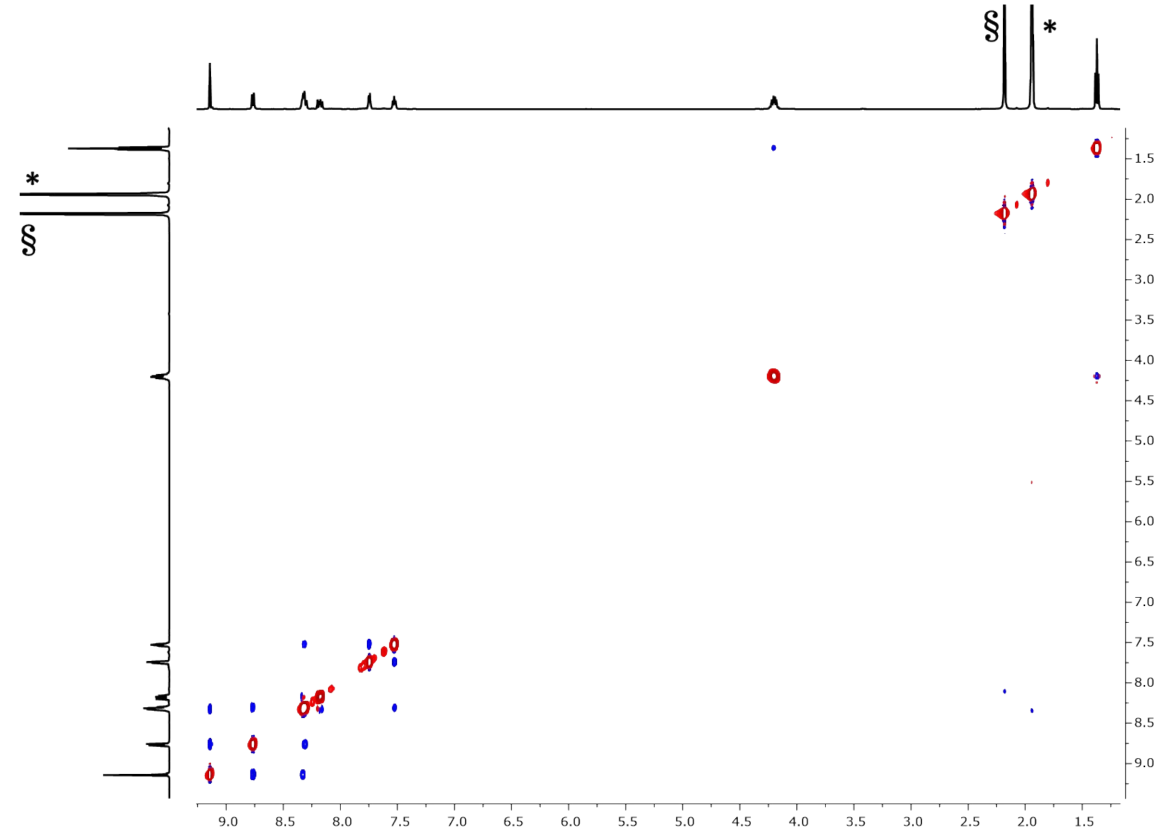

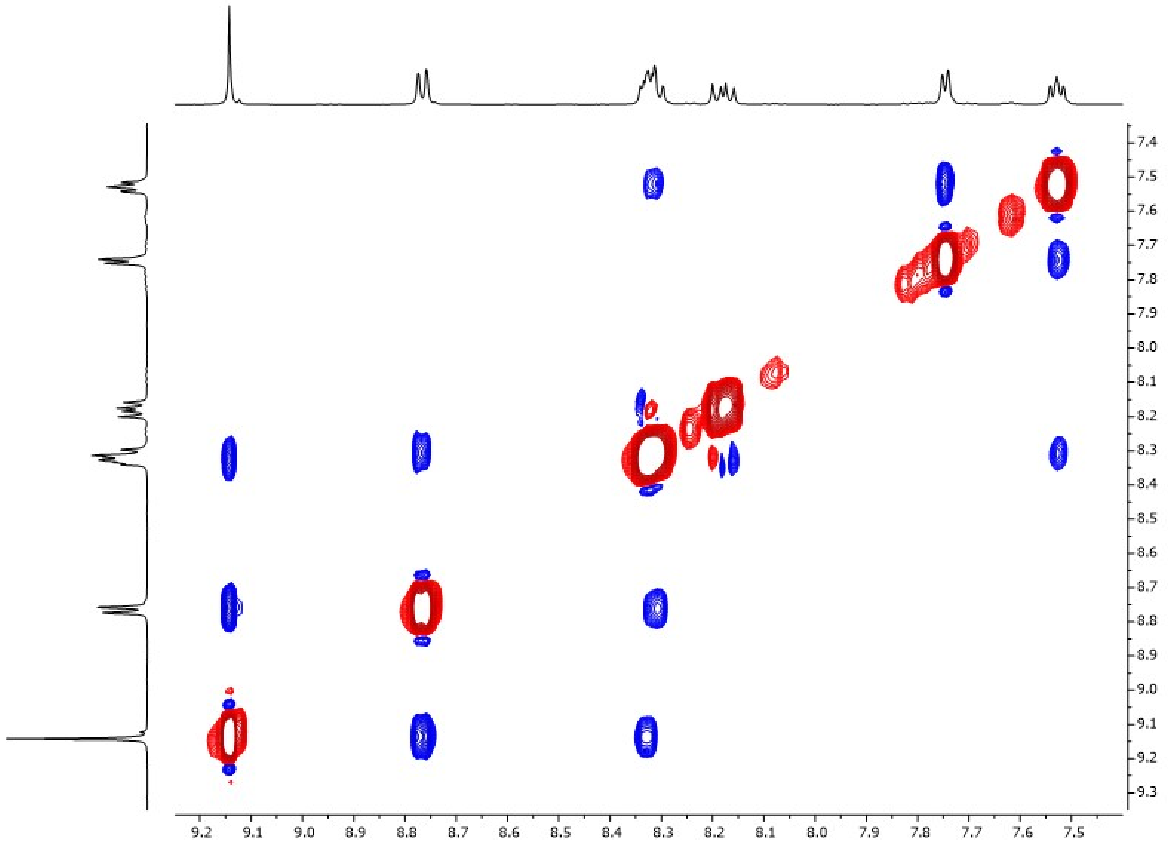

Fig. S17. NOESY (500 MHz, CD_3CN, 298 K) spectrum of [Rh(**5**)$_2$][PF$_6$]$_3$, * = CHD_2CN, § = HOD. Chemical shifts in δ/ppm.

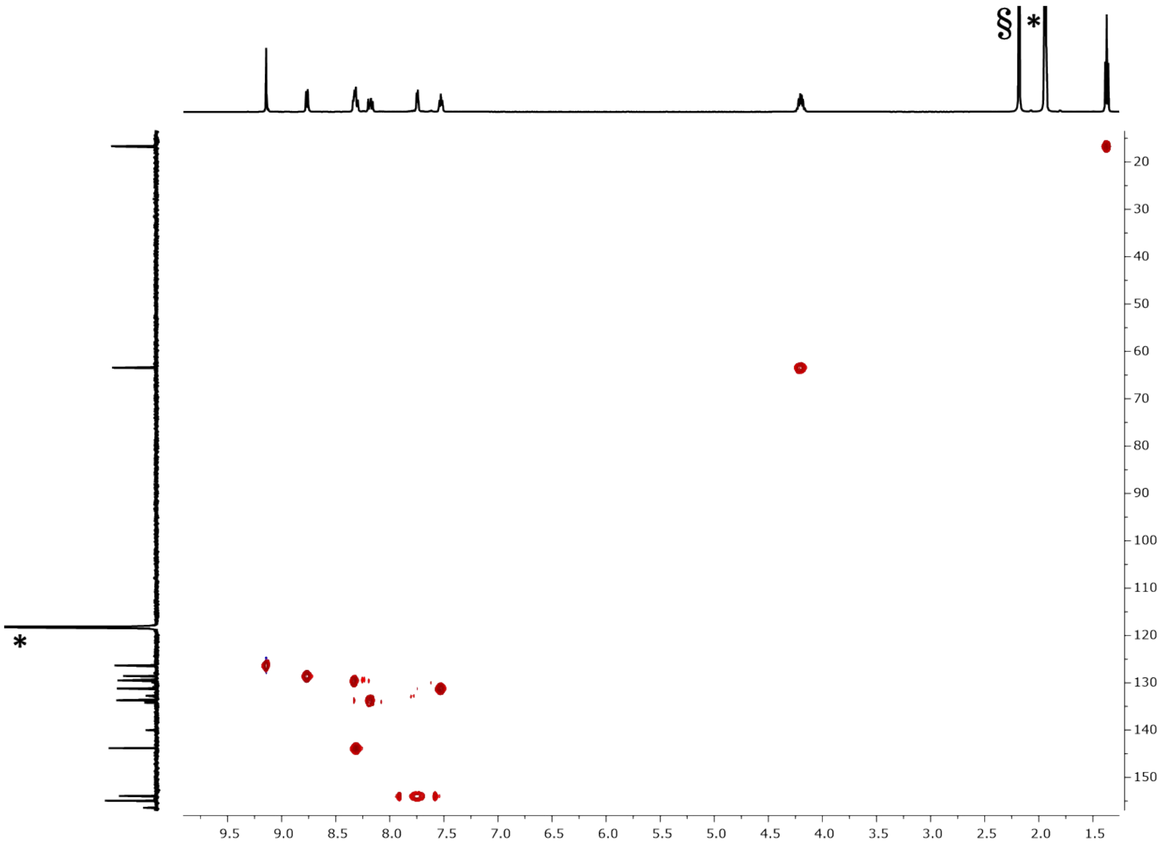

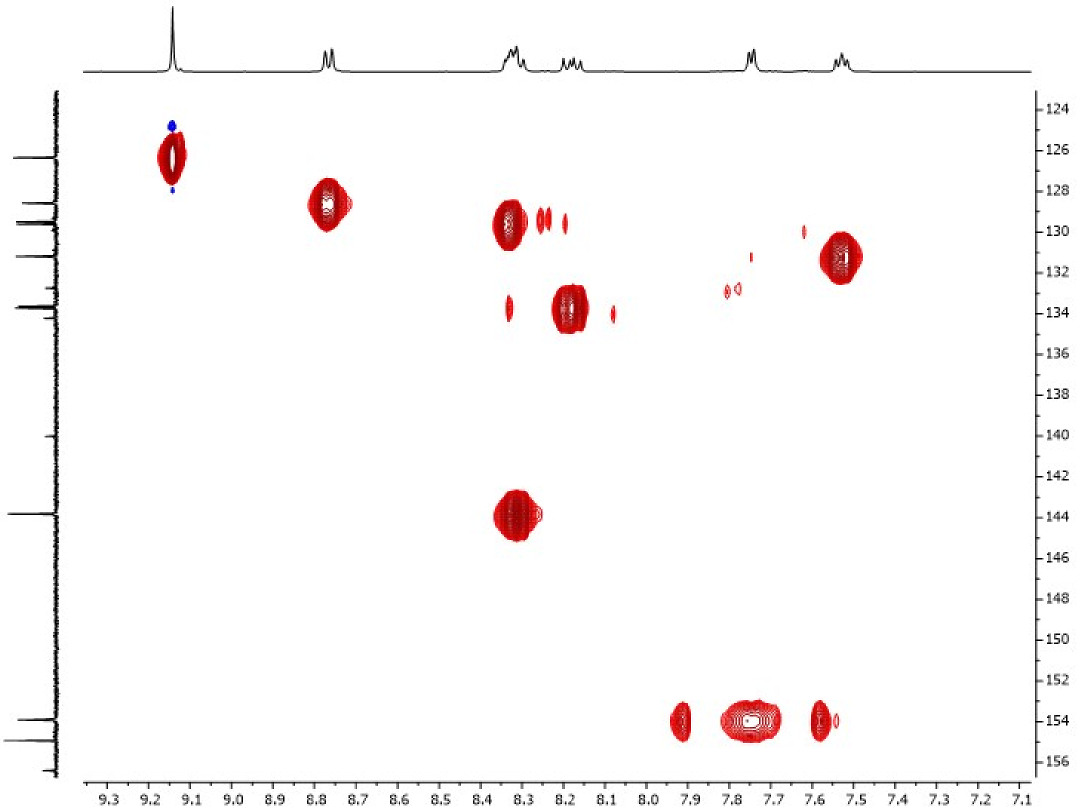

Fig. S18. HMQC (500 MHz ^{1}H, 126 MHz ^{13}C, CD_3CN, 298 K) spectrum of [Rh(**5**)$_2$][PF$_6$]$_3$, * = CHD_2CN, § = HOD. Chemical shifts in δ/ppm.

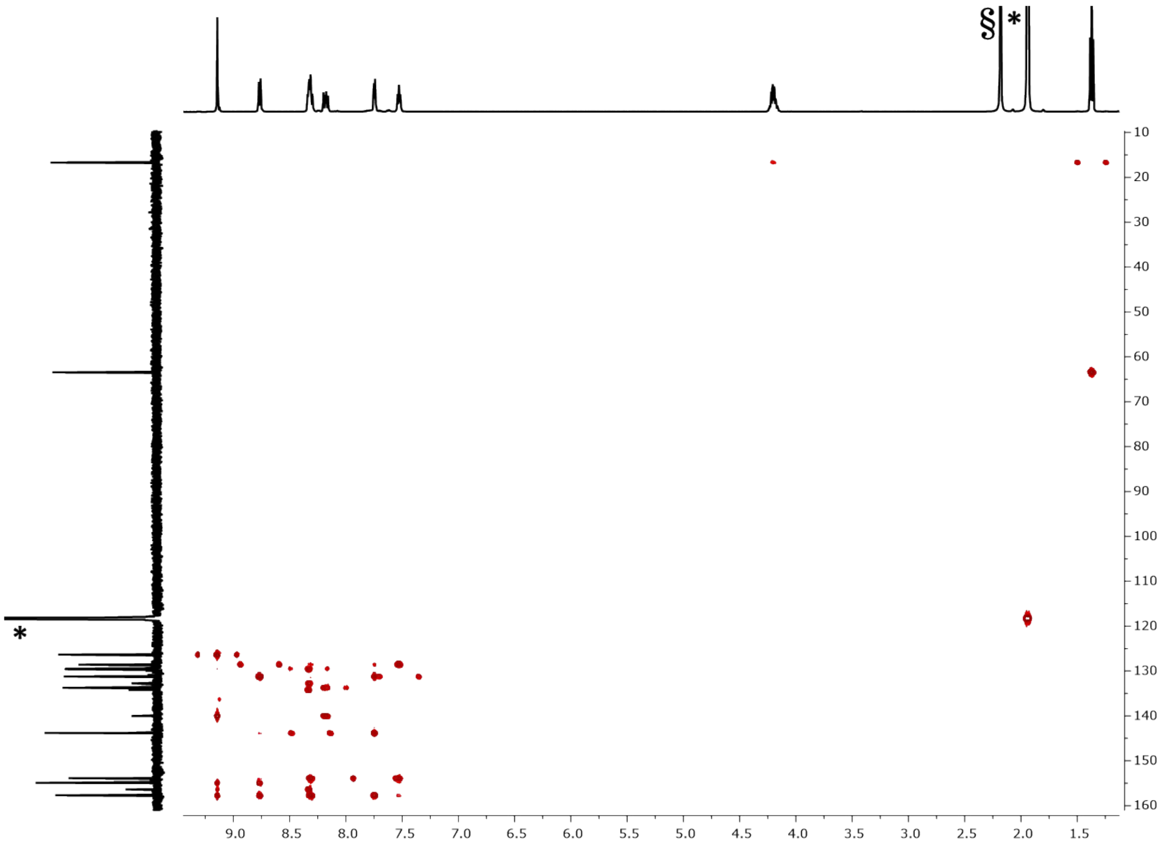

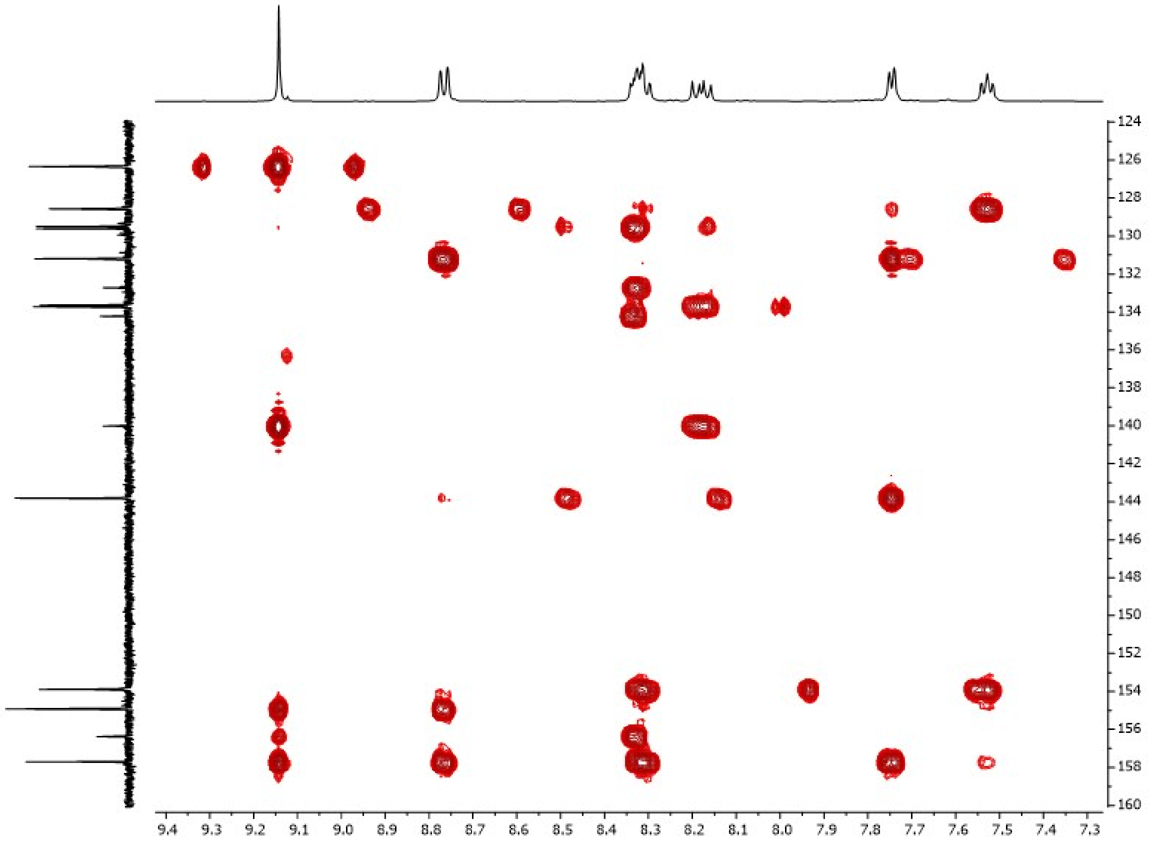

Fig. S19. HMBC (500 MHz ^{1}H, 126 MHz ^{13}C, CD_3CN, 298 K) spectrum of $[Rh(\mathbf{5})_2][PF_6]_3$, * = CHD_2CN, § = HOD. Chemical shifts in δ/ppm.

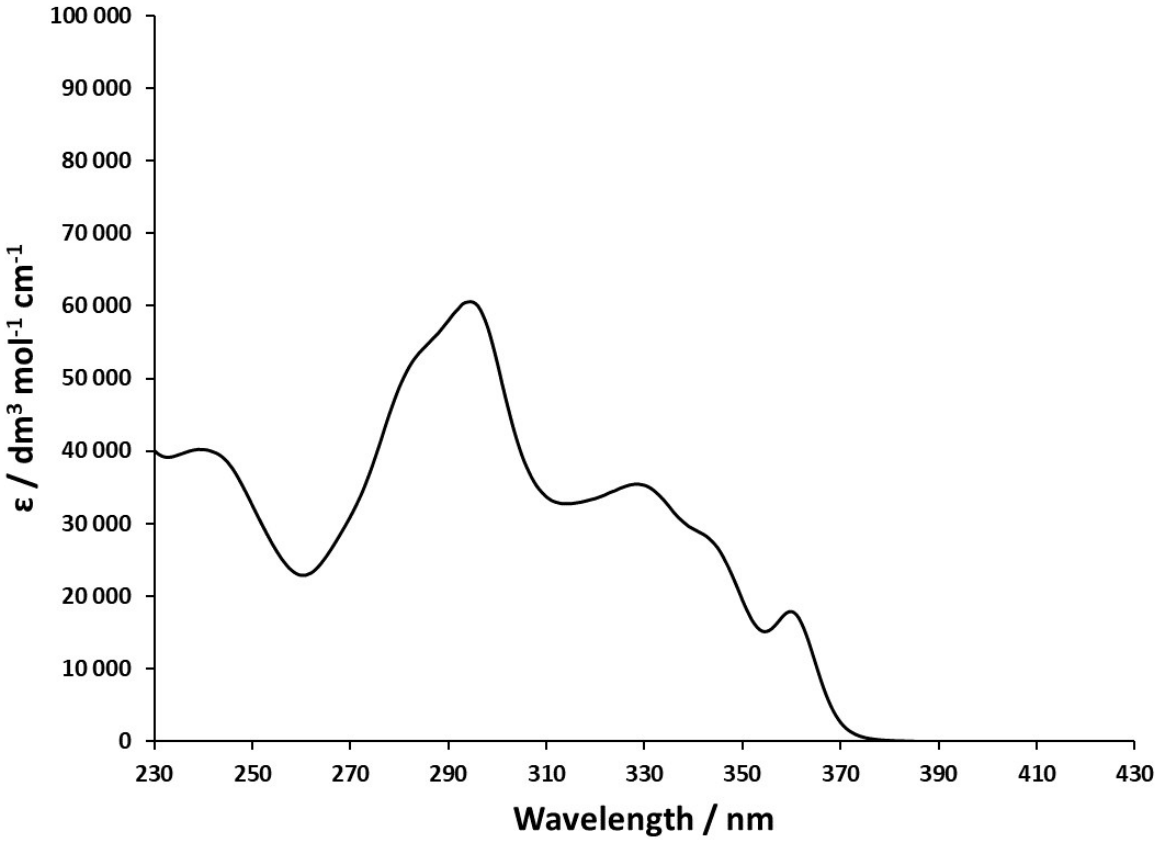

Fig. S20. Solution absorption spectra of $[Rh(\mathbf{5})_2][PF_6]_3$ (2.25×10^{-5} mol dm^{-3}) in MeCN.

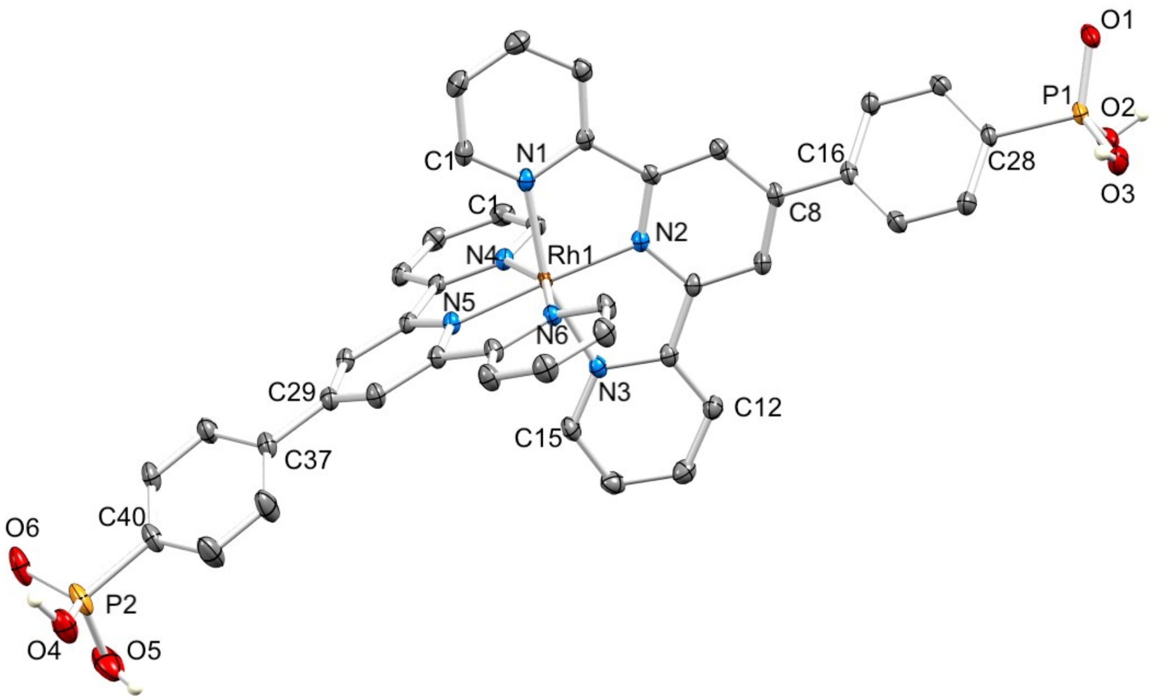

(a)

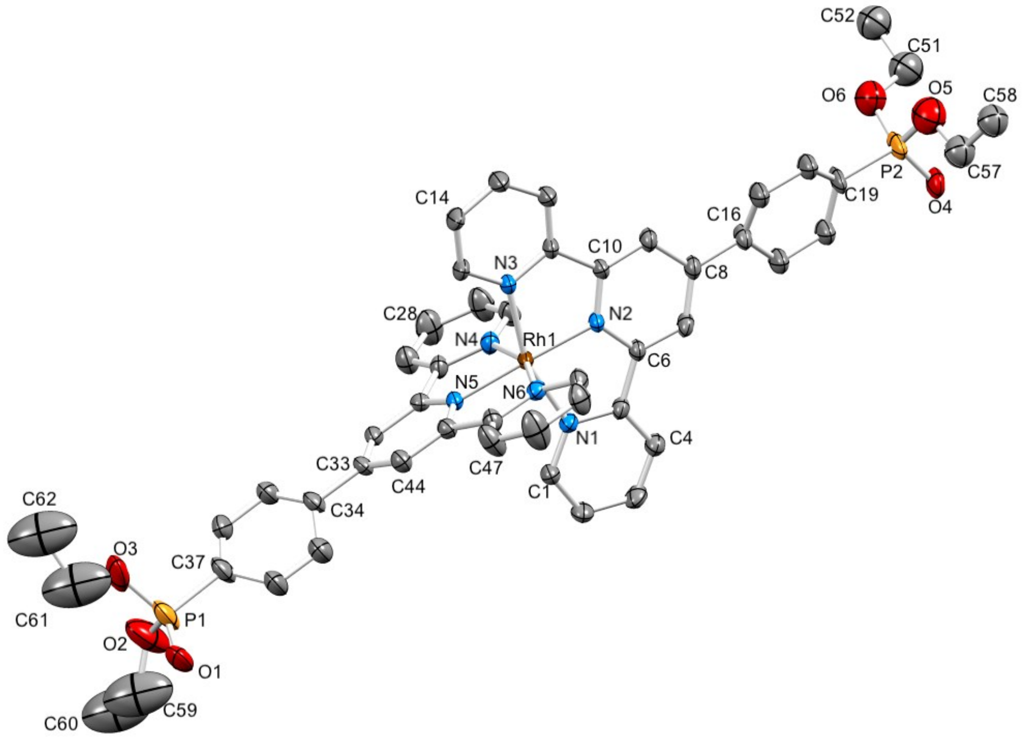

(b)

Fig. S21. (a) An ORTEP representation of the structure of the $[Rh(\mathbf{1})_2]^{3+}$ cation in $[Rh(\mathbf{1})_2][NO_3]_3{\cdot}1.25[H_3O][NO_3]{\cdot}2.75H_2O$ with ellipsoids plotted at 50% probability level. H atoms except fro those attached to the phosphonic acid groups are omitted for clarity. (b) An ORTEP representation of the structure of the $[Rh(\mathbf{5})_2]^{3+}$ cation in $[Rh(\mathbf{5})_2][PF_6]_3{\cdot}MeCN$ with ellipsoids plotted at 30% probability level and H atoms omitted for clarity. Both phenylene rings and the $PO(OEt)_2$ group including P2 were disordered (see Experimental section for details).

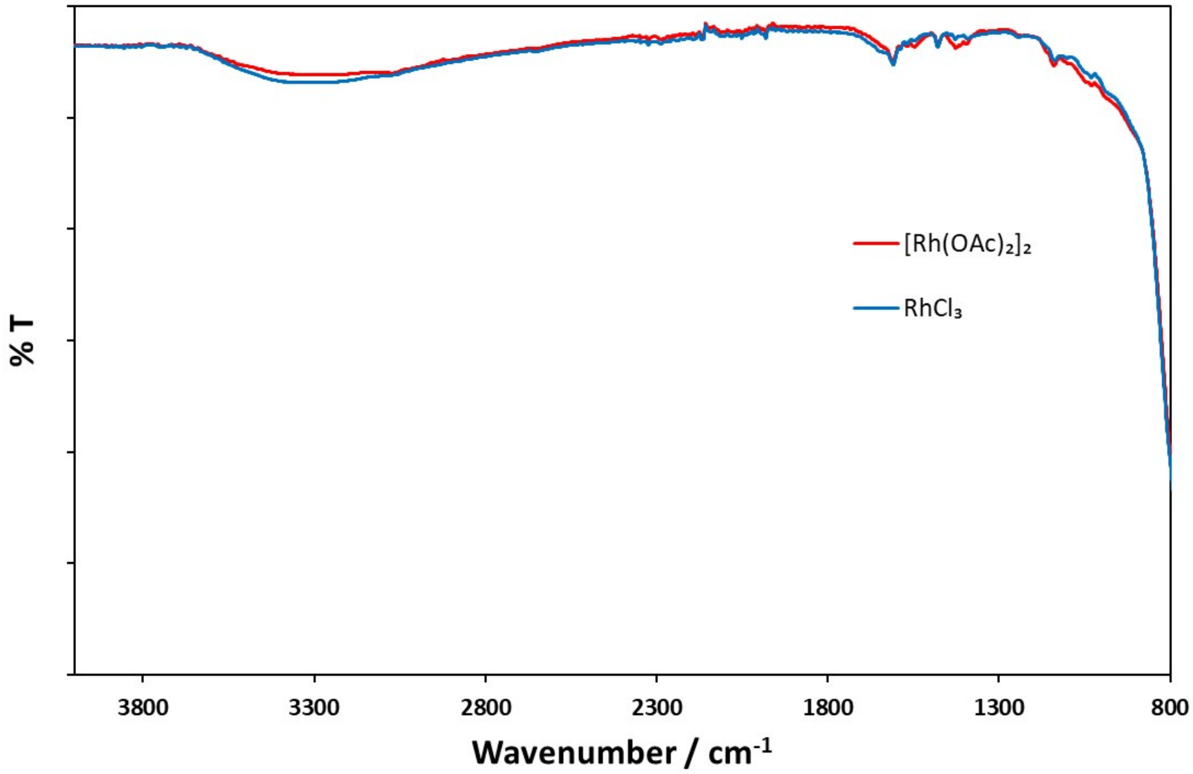

Fig. S22. Solid-state IR spectra of NP-[Rh(**1**)$_2$]$^{3+}$, prepared from [Rh_2(μ-OAc)$_4$(H_2O)$_2$] (red) or $RhCl_3.3H_2O$ (blue) following the procedures in the Experimental Section.

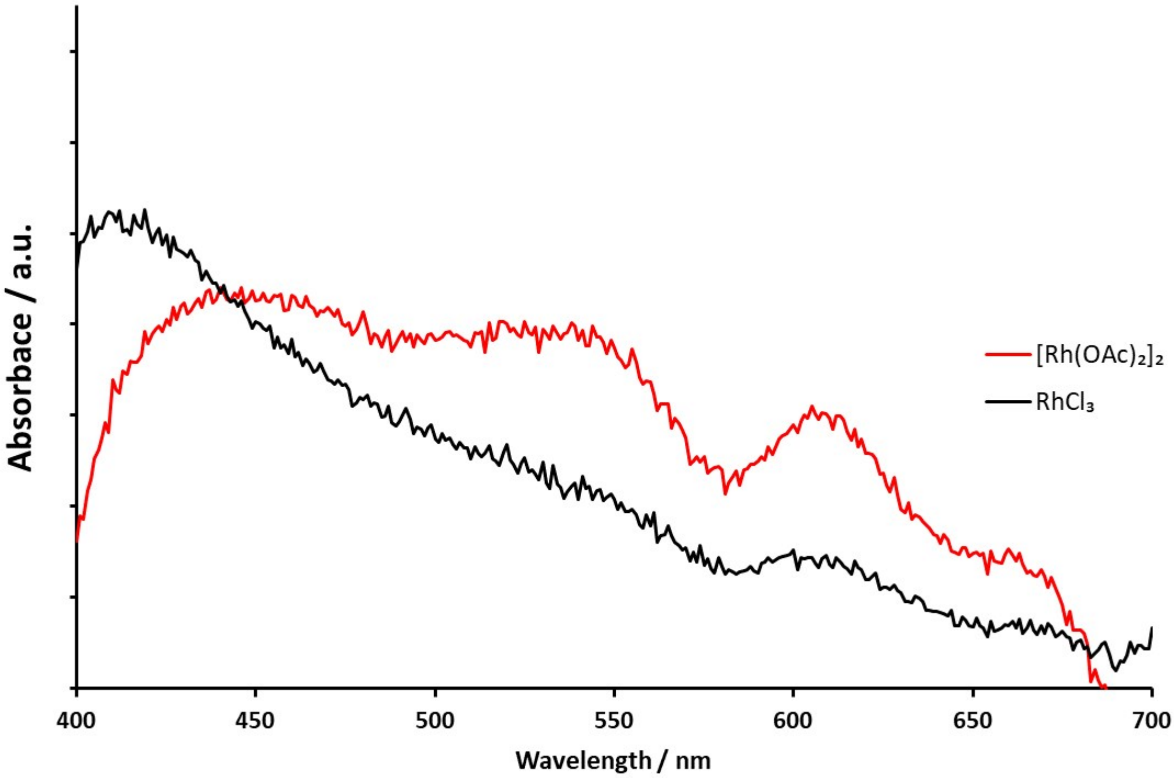

Fig. S23. Solid-state absorption spectra of NP-[Rh(**1**)$_2$]$^{3+}$ prepared from [Rh_2(μ-OAc)$_4$(H_2O)$_2$] (red) or $RhCl_3.3H_2O$ (black); The spectra are background-corrected using an NP reference.

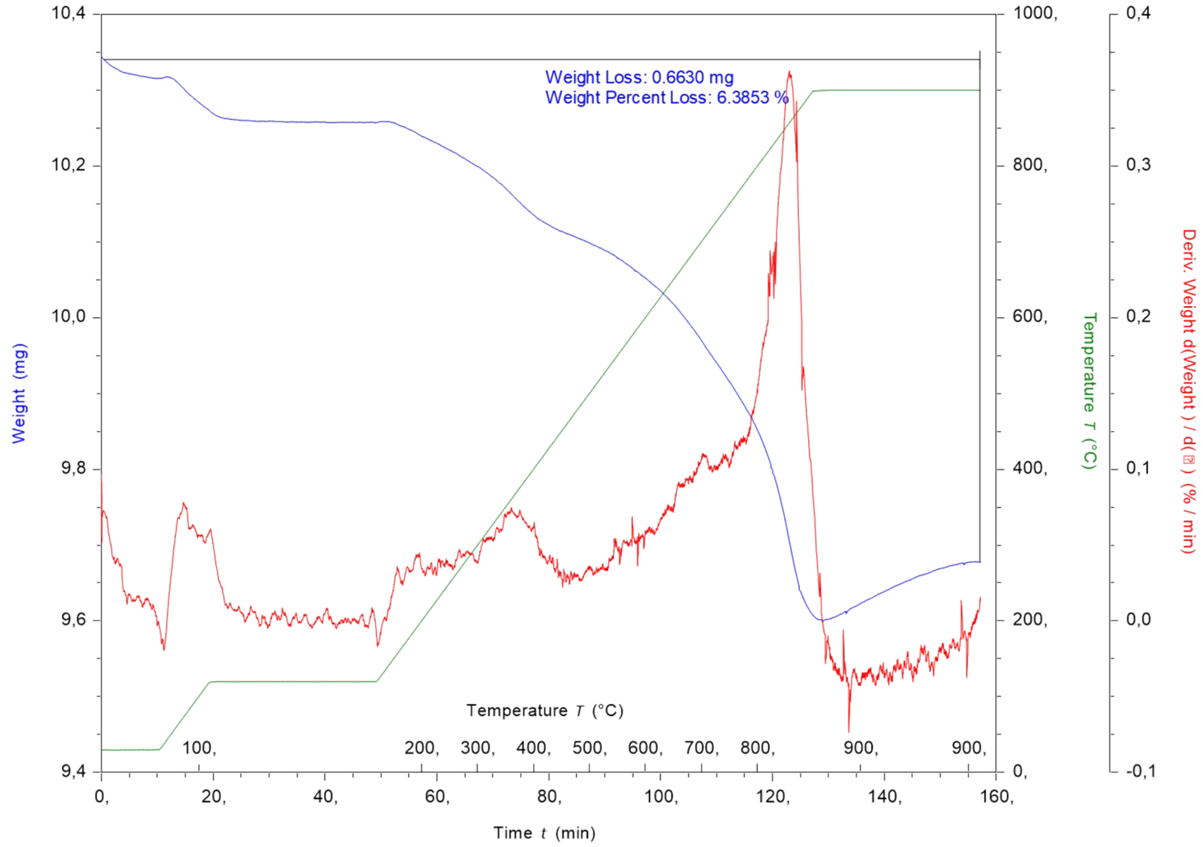

Fig. S24. TGA curve for NP-[Rh(**1**)$_2$]$^{3+}$ prepared from [Rh$_2$(μ-OAc)$_4$(H$_2$O)$_2$]: Weight against time and temperature (blue), derivative of weight change against time (red).

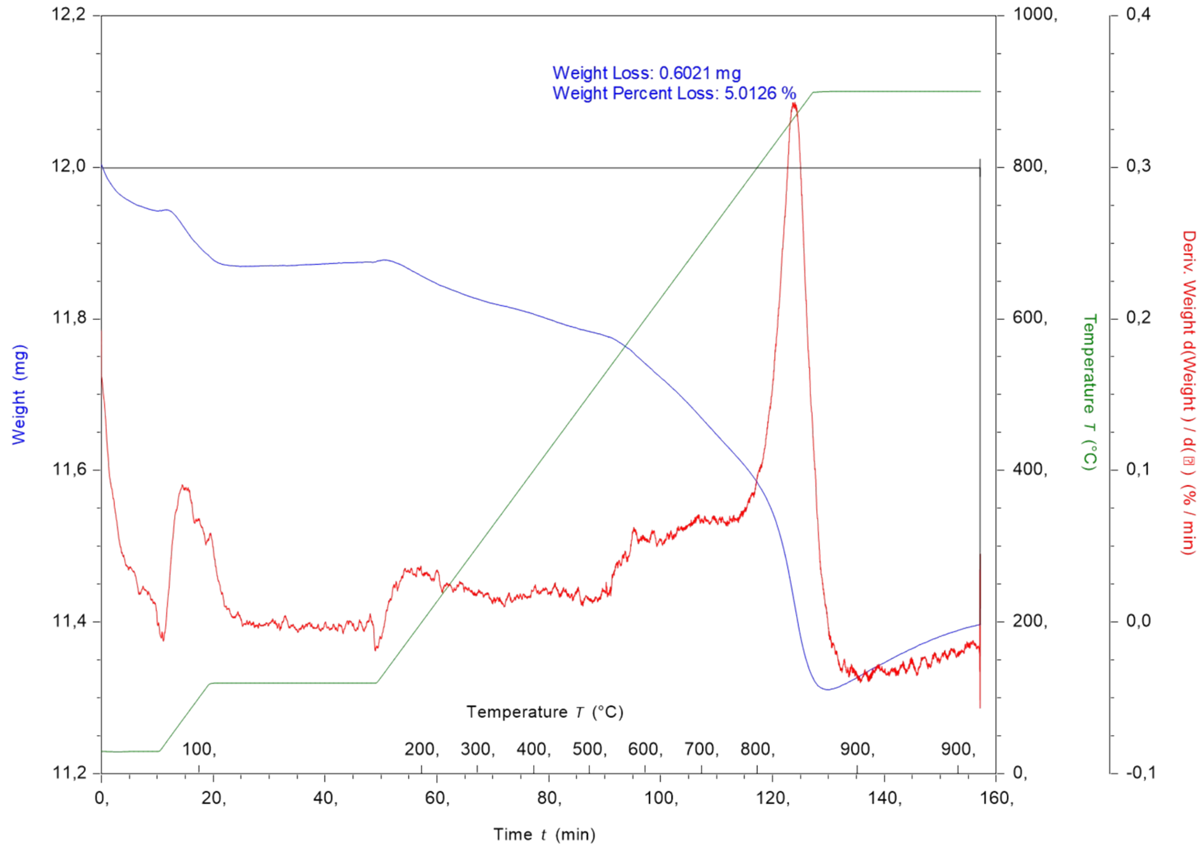

Fig. S25. TGA curve for NP-[Rh(**1**)$_2$]$^{3+}$ prepared from RhCl$_3$.3H$_2$O: Weight against time and temperature (blue), derivative of weight change against (red).

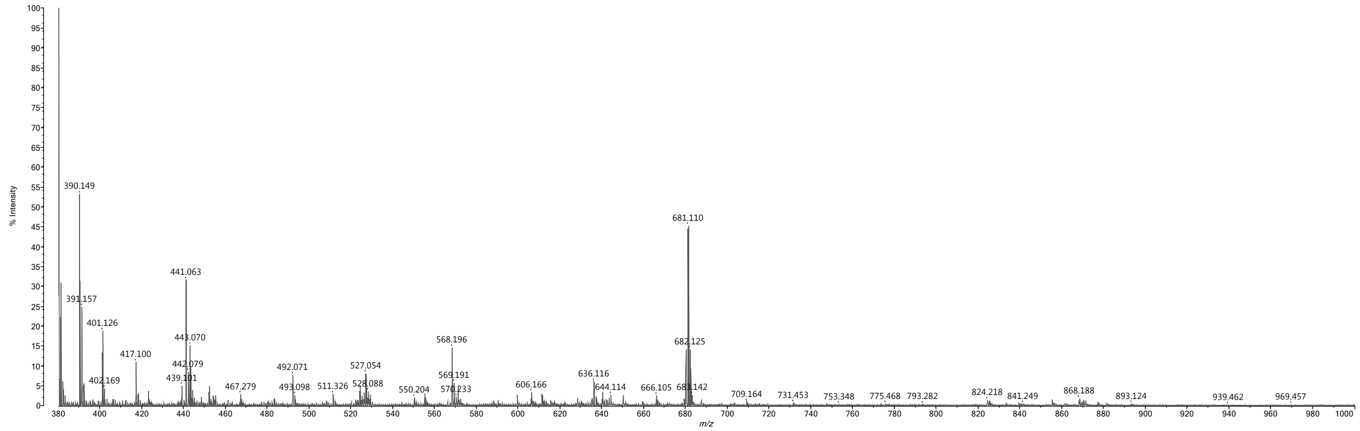

Fig. S26. MALDI mass spectrum (with CHCA matrix) of (**1**)@TiO_2 NPs prepared from $[Rh_2(\mu\text{-}OAc)_4(H_2O)_2]$.

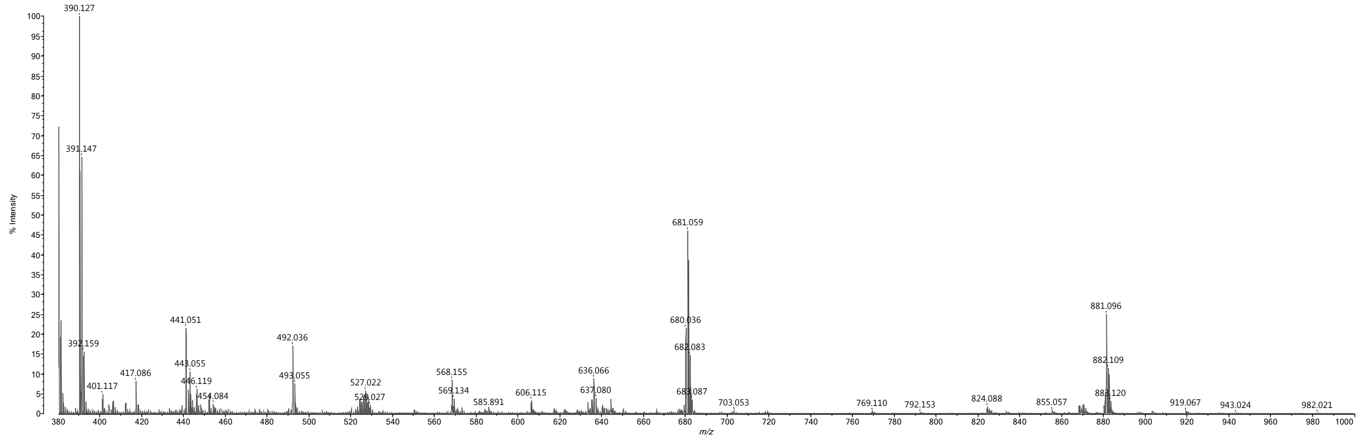

Fig. S27. MALDI mass spectrum (with CHCA matrix) of Rh(**1**)$_2$@TiO_2 NPs prepared from $RhCl_3.3H_2O$.

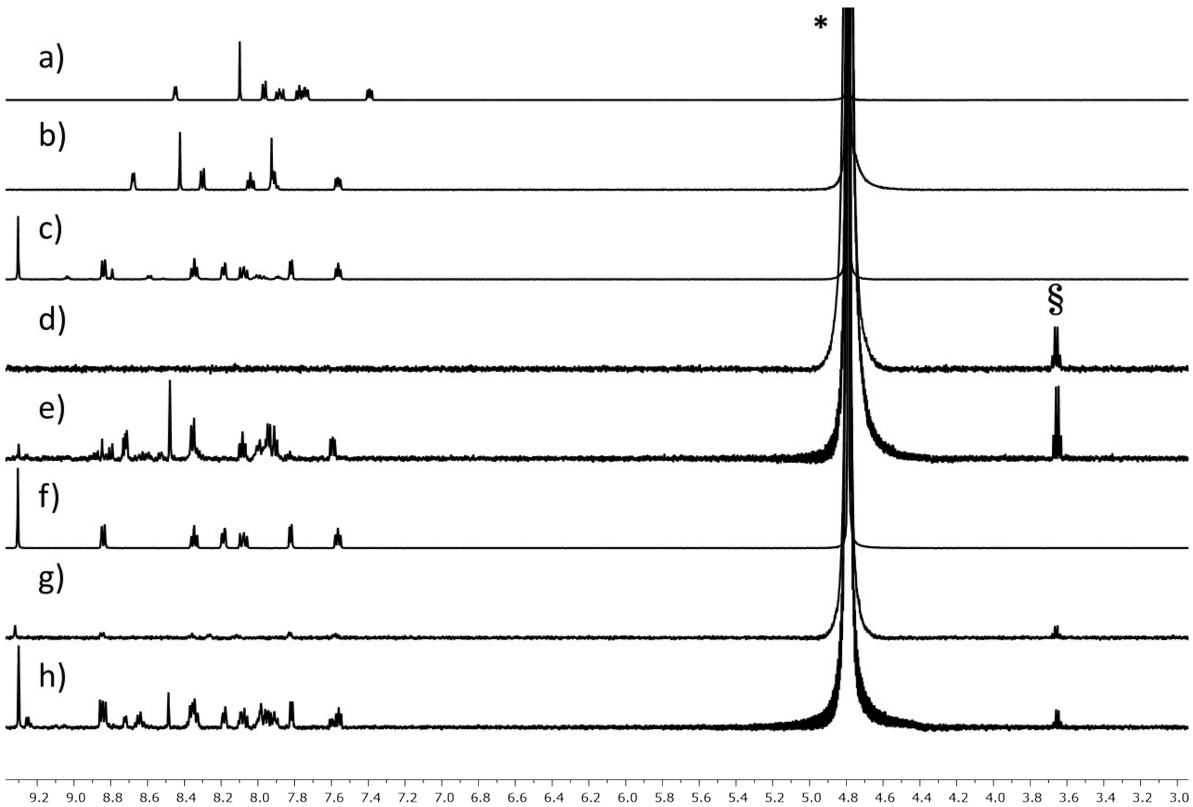

Fig. S28. ^{1}H NMR spectra (500 MHz, D_2O, 298 K) of **1** in D_2O and NaOH (a), (**1**)@TiO_2 in D_2O and NaOH (b), [Rh(**1**)$_2$]Cl_3 made using [Rh_2(μ-OAc)$_4$(H_2O)$_2$] in D_2O and NaOH (c), Rh(**1**)$_2$@TiO_2 NPs made using [Rh_2(μ-OAc)$_4$(H_2O)$_2$] in D_2O (d), Rh(**1**)$_2$@TiO_2 NPs made using [Rh_2(μ-OAc)$_4$(H_2O)$_2$] in D_2O and NaOH (e), [Rh(**1**)$_2$]Cl_3 made using $RhCl_3$.3H_2O in D_2O and NaOH (f), Rh(**1**)$_2$@TiO_2 NPs made using $RhCl_3$.3H_2O in D_2O (g), Rh(**1**)$_2$@TiO_2 NPs made using $RhCl_3$.3H_2O in D_2O and NaOH (h). Chemical shifts in δ/ppm.

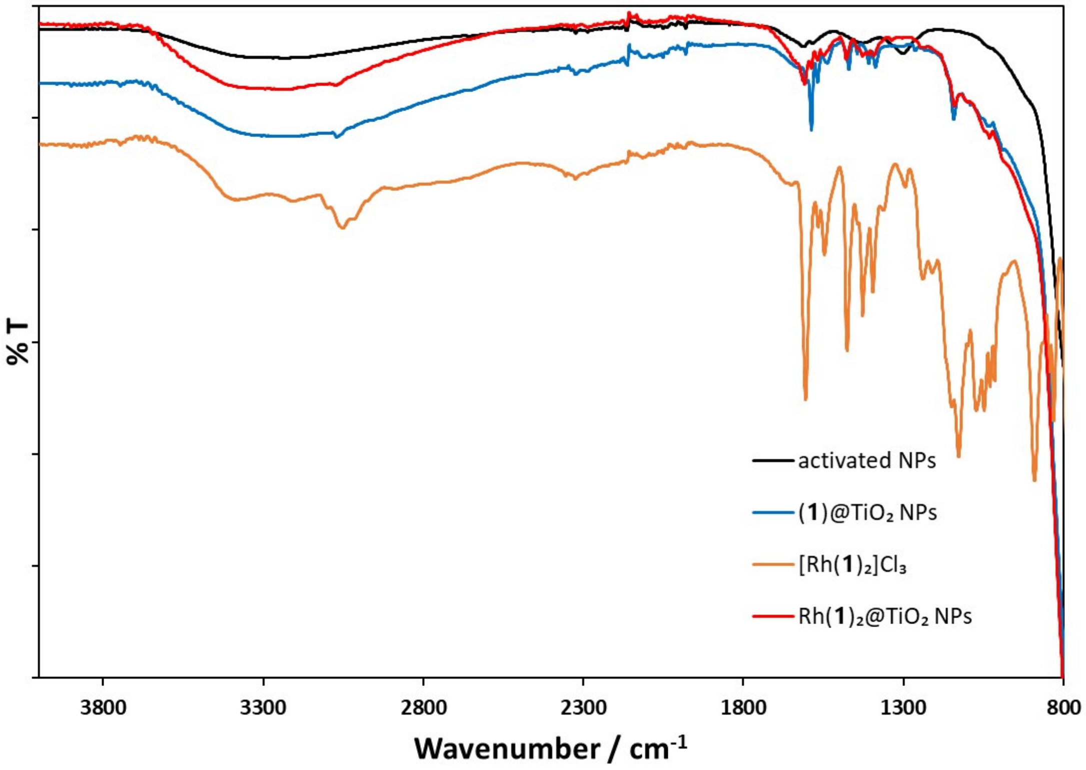

Fig. S29. Solid-state IR spectra of activated NPs (black), (**1**)@TiO_2 NPs (blue), [Rh(**1**)$_2$]Cl_3 (orange) and Rh(**1**)$_2$@TiO_2 NPs (red) following the procedures in the Experimental Section.

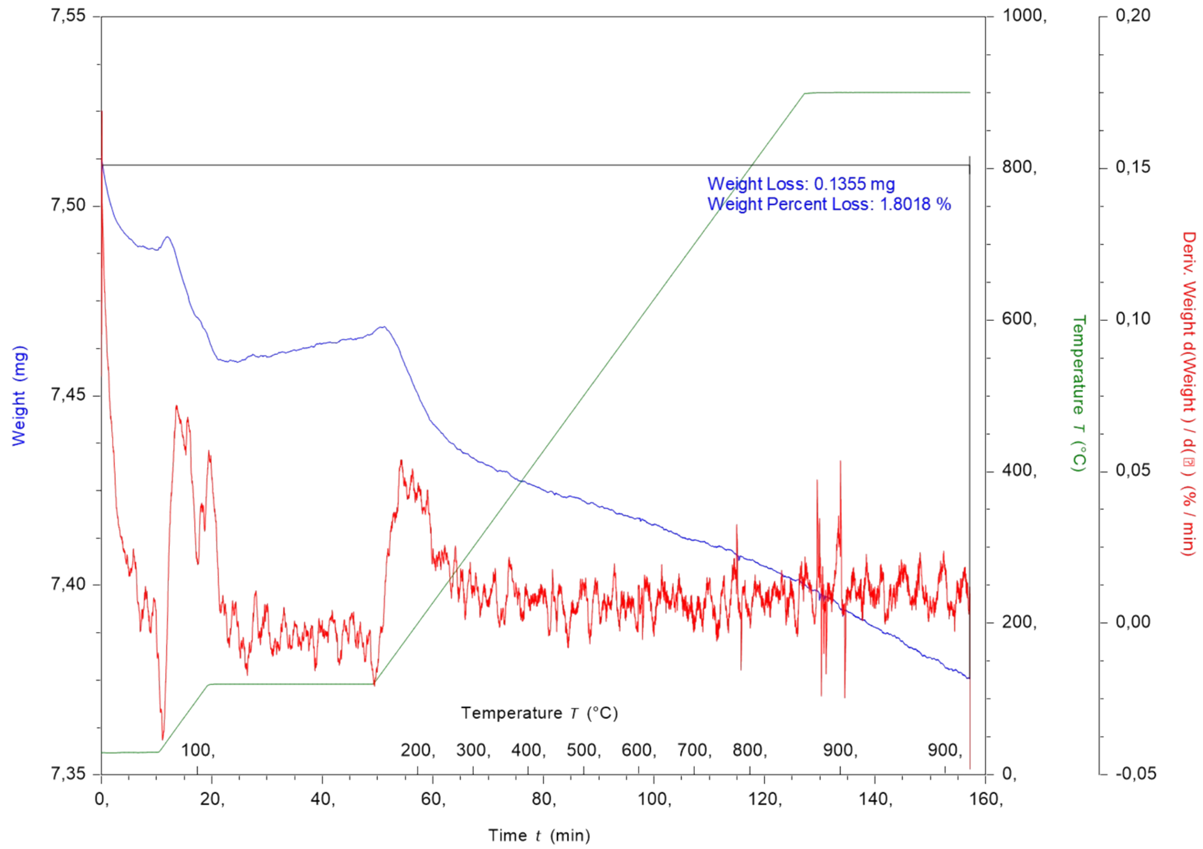

Fig. S30. TGA curve for commercial NPs: Weight against time and temperature (blue), derivative of weight change against time (red).

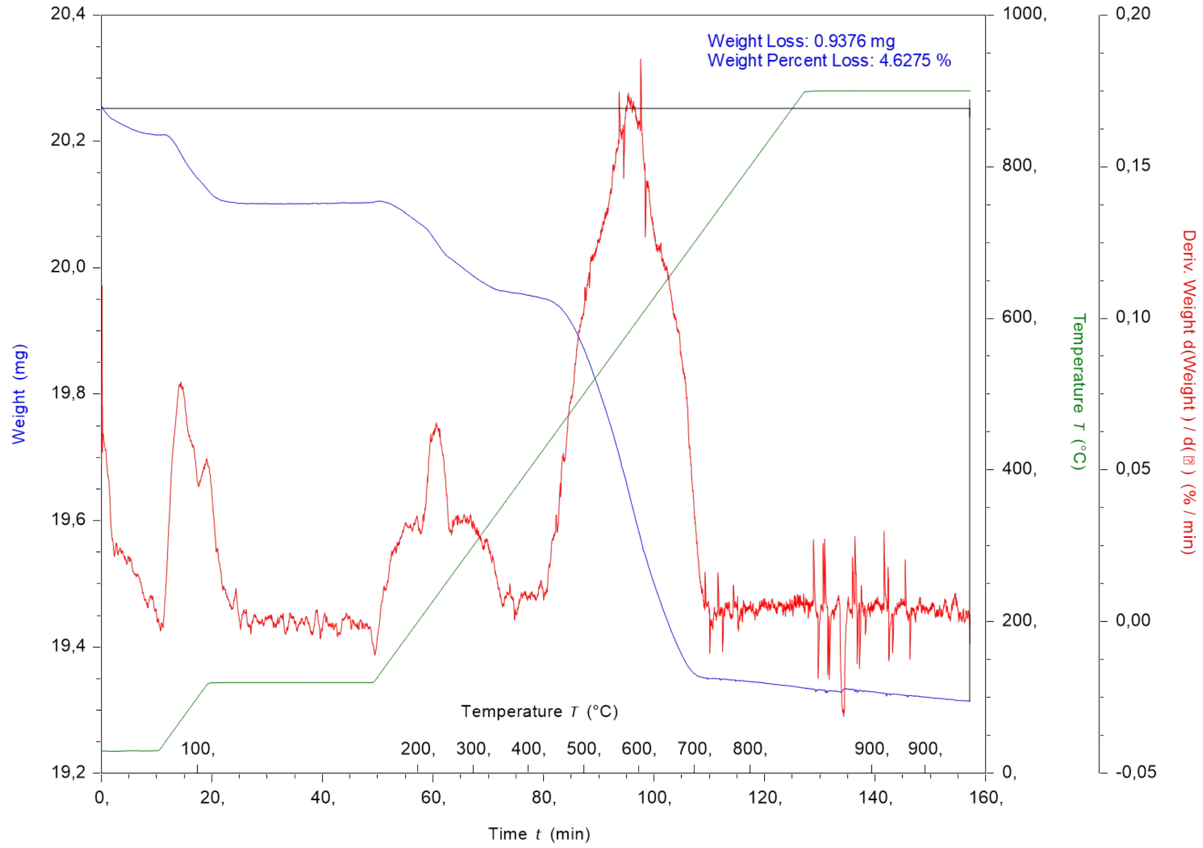

Fig. S31. TGA curve of (**1**)@TiO_2 NPs: Weight against time and temperature (blue), derivative of weight change against time (red).

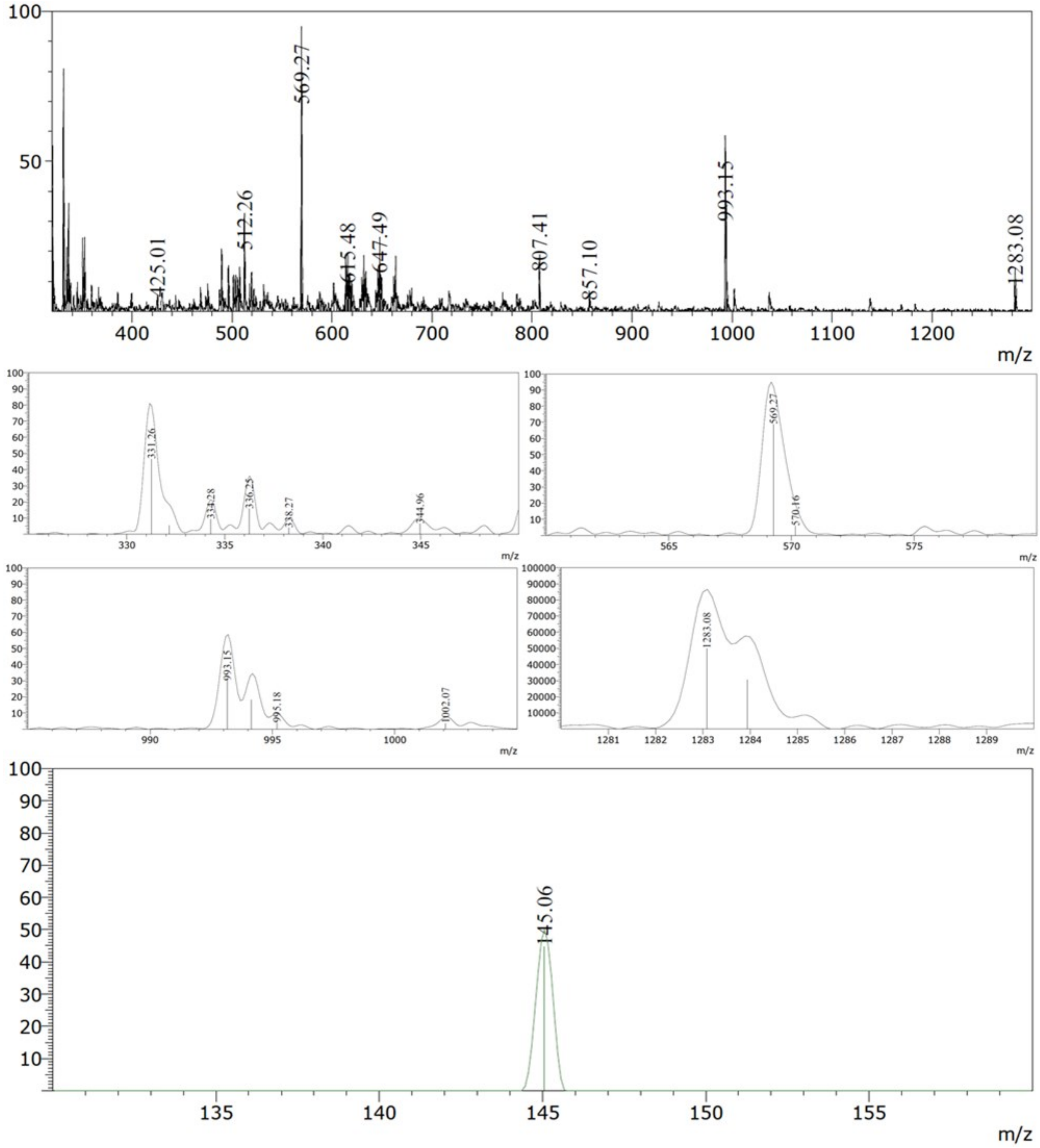

Fig. S32. Electrospray mass spectrum of [Rh(**5**)$_2$][PF$_6$]$_3$ (MeCN). Positive mode in black, negative mode in green.

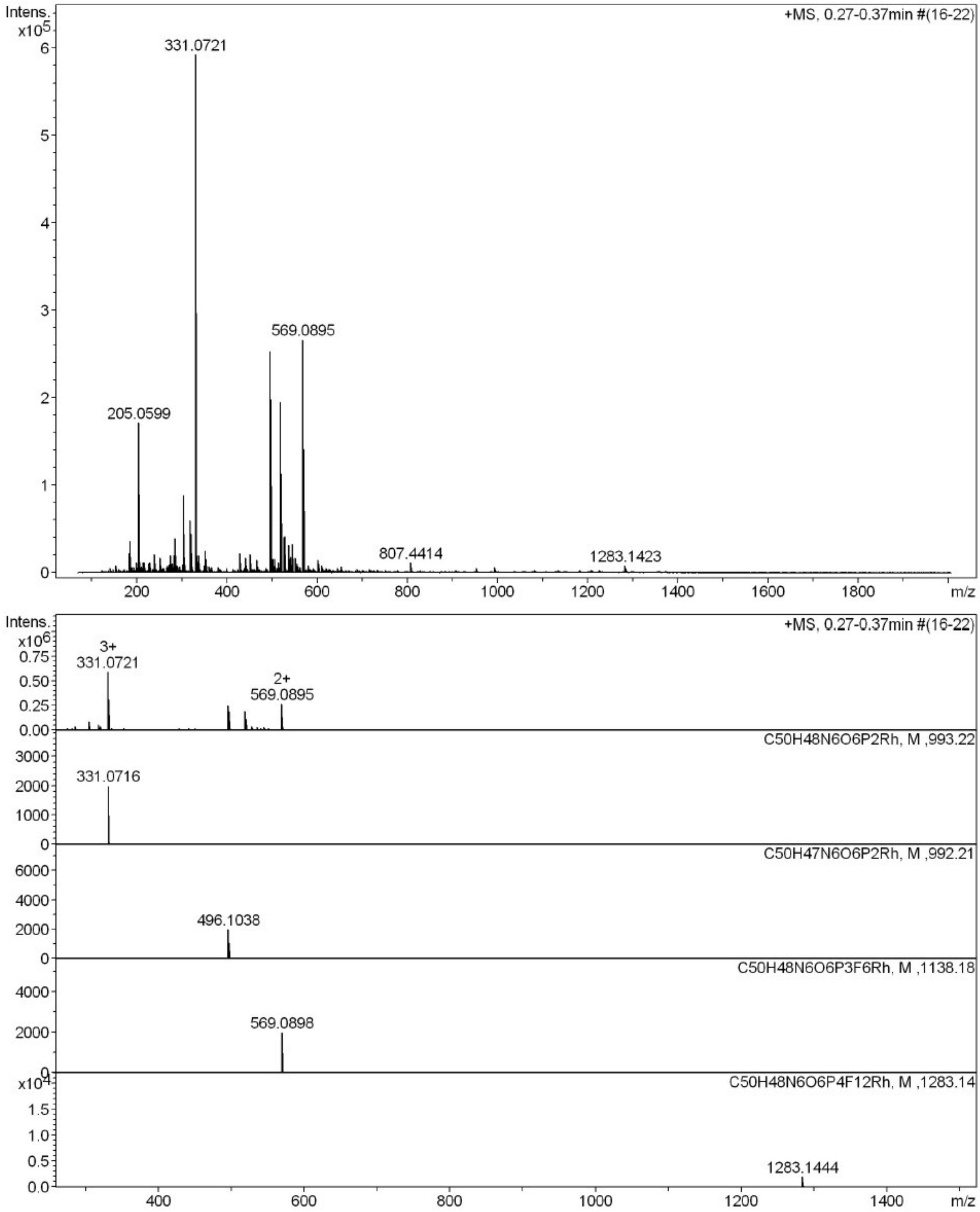

Measured m/z vs. theoretical m/z

Meas. m/z	#	Formula	Score	m/z	err [mDa]	err [ppm]	mSigma	rdb	e⁻ Conf	z
331.0721	1	C 50 H 48 N 6 O 6 P 2 Rh	100.00	331.0716	-0.5	-1.4	14.4	31.5	even	3+
496.1035	1	C 50 H 47 N 6 O 6 P 2 Rh	100.00	496.1038	0.3	0.6	126.5	32.0	even	2+
569.0895	1	C 50 H 48 F 6 N 6 O 6 P 3 Rh	100.00	569.0898	0.3	0.5	19.2	29.0	even	
1283.1423	1	C 50 H 48 F 12 N 6 O 6 P 4 Rh	100.00	1283.1444	2.1	1.7	40.2	26.5	even	1+

Fig. S33. High resolution electrospray mass spectrum of $[Rh(\mathbf{5})_2][PF_6]_3$ (MeCN, positive mode). Calculated spectra are shown in the lower four traces.

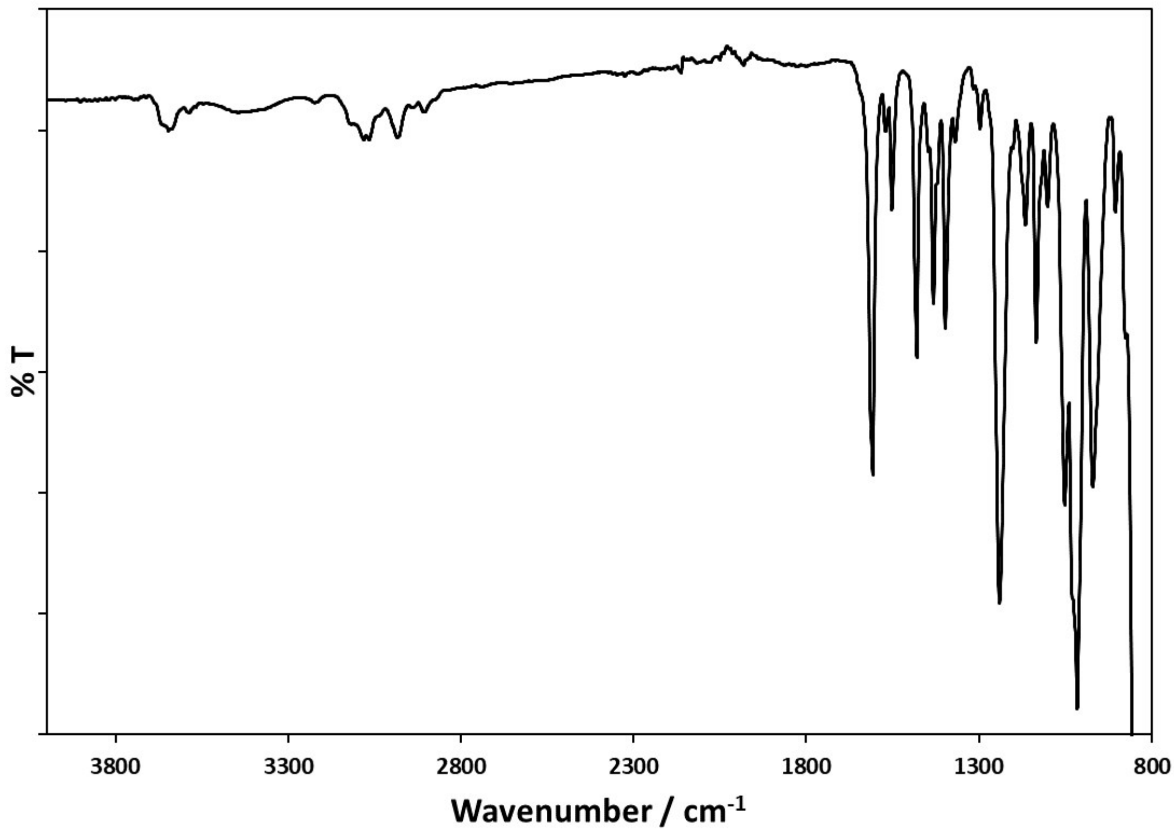

Fig. S34. Solid-state IR spectrum of [Rh(**5**)$_2$][PF$_6$]$_3$.

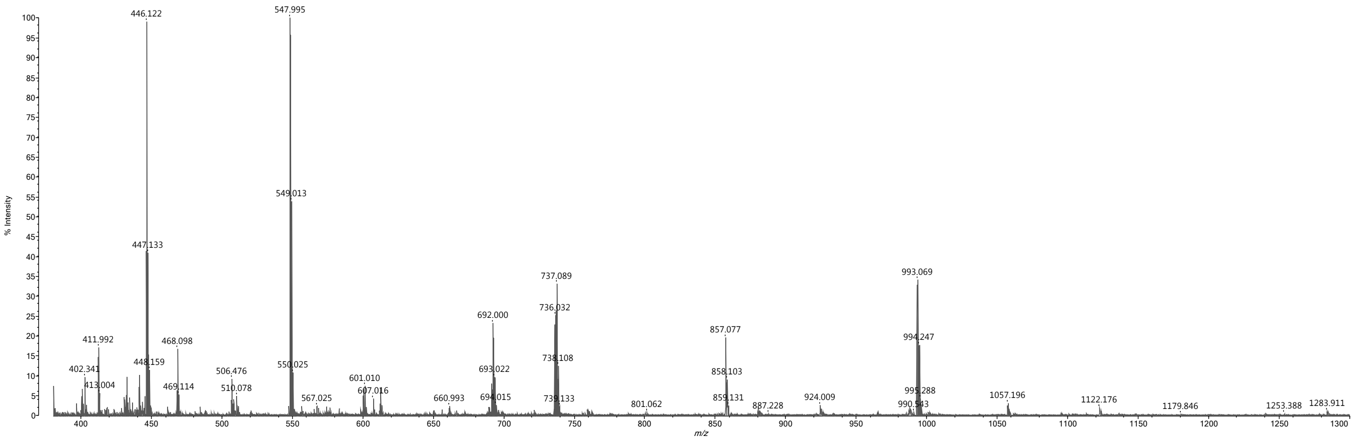

Fig. S35. MALDI mass spectrum (with CHCA matrix) of [Rh(**5**)$_2$][PF$_6$]$_3$

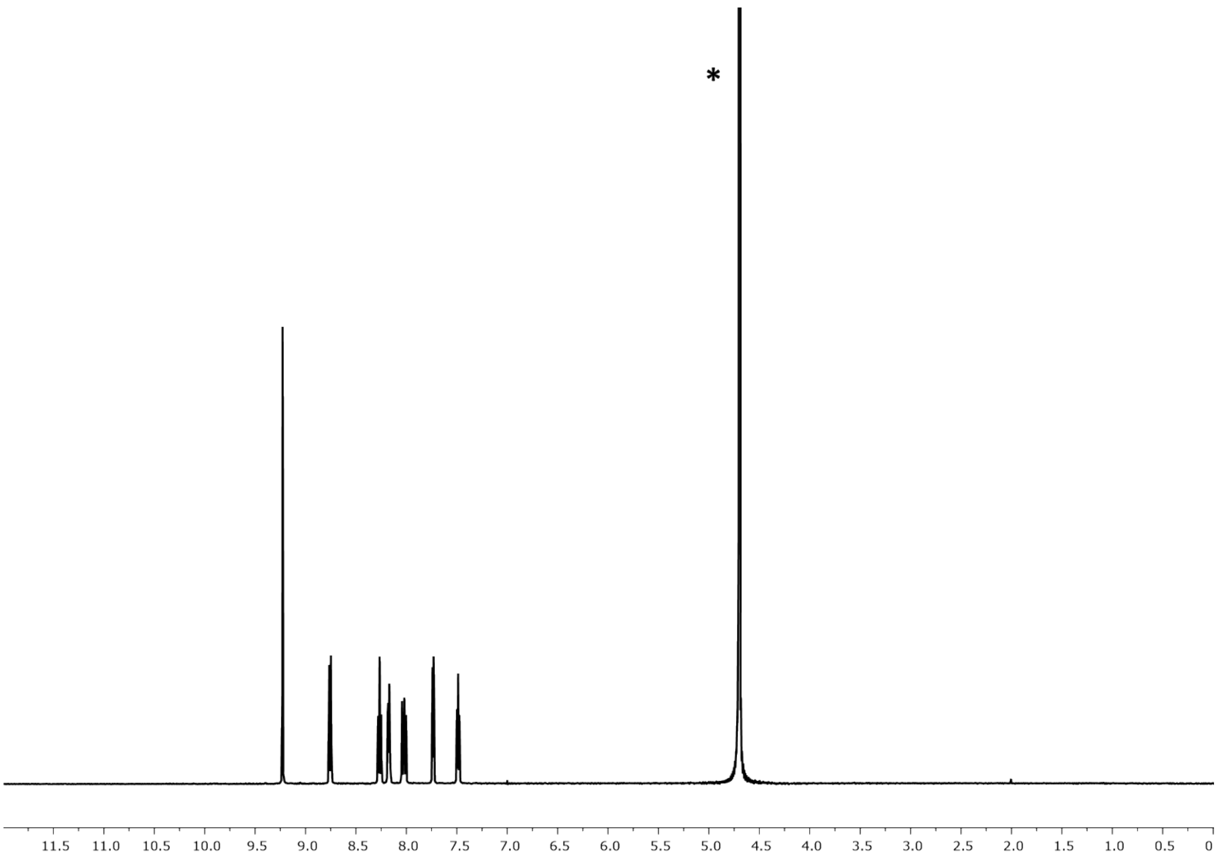

Fig. S36. ^{1}H NMR (500 MHz, D_2O, 298 K) spectrum of $[Rh(\mathbf{1})_2]Cl_3$ prepared from $RhCl_3.3H_2O$, * = HOD. Chemical shifts in δ/ppm.

Table S1: Conditions and yields for the oxidation of *rac*-(1*R*)-1-phenylethanol to acetophenone.

**Conditions**	**Yield (Product)**
$Rh(\mathbf{1})_2@TiO_2$ NPs (0.5 mol%), NaOH (25 mM), 100 °C, 24 h	23.2%
$Rh(\mathbf{1})_2@TiO_2$ NPs (0.5 mol%), NaOH (50 mM), 100 °C, 24 h	21.9%
$Rh(\mathbf{1})_2@TiO_2$ NPs (0.5 mol%), NaOH (0.25 M), 100 °C, 24 h	21.9%
$Rh(\mathbf{1})_2@TiO_2$ NPs (0.5 mol%), NaOH (2.5 M), 100 °C, 24 h	18.7%
$Rh(\mathbf{1})_2@TiO_2$ NPs (0.5 mol%), NaOH (25 mM), 22 °C, 72 h	< 1%
Commercial NPs (0.5 mol%), NaOH (25 mM), 100 °C, 24 h	< 1%
Activated NPs (0.5 mol%), NaOH (25 mM), 100 °C, 24 h	< 1%
Activated NPs (0.5 mol%), $[Rh_2(\mu\text{-}OAc)_4(H_2O)_2]$ (0.5 mol%), NaOH (25 mM), 100 °C, 24 h	< 1%
$(\mathbf{1})@TiO_2$ NPs (0.5 mol%), NaOH (25 mM), 100 °C, 24 h	< 1%
$(\mathbf{1})@TiO_2$ NPs (0.5 mol%), $[Rh_2(\mu\text{-}OAc)_4(H_2O)_2]$ (0.5 mol%), NaOH (25 mM), 100 °C, 24 h	7%
$[Rh(\mathbf{1})_2]Cl_3$ (0.5 mol%), NaOH (25 mM), 100 °C, 24 h	15.8%
$[Rh(\mathbf{5})_2][PF_6]_3$ (0.5 mol%), NaOH (25 mM), 100 °C, 24 h	19.2%
$Rh(\mathbf{1})_2@TiO_2$ NPs (0.5 mol%), NaOH (25 mM), 100 °C, 72 h, air	29.3%
$Rh(\mathbf{1})_2@TiO_2$ NPs (0.5 mol%), NaOH (25 mM), 100 °C, 72 h, argon	16.5%
